# A high-performance genetically encoded fluorescent indicator for in vivo cAMP imaging

**DOI:** 10.1038/s41467-022-32994-7

**Published:** 2022-09-12

**Authors:** Liang Wang, Chunling Wu, Wanling Peng, Ziliang Zhou, Jianzhi Zeng, Xuelin Li, Yini Yang, Shuguang Yu, Ye Zou, Mian Huang, Chang Liu, Yefei Chen, Yi Li, Panpan Ti, Wenfeng Liu, Yufeng Gao, Wei Zheng, Haining Zhong, Shangbang Gao, Zhonghua Lu, Pei-Gen Ren, Ho Leung Ng, Jie He, Shoudeng Chen, Min Xu, Yulong Li, Jun Chu

**Affiliations:** 1 Research Center for Biomedical Optics and Molecular Imaging, Shenzhen Key Laboratory for Molecular Imaging, Guangdong Provincial Key Laboratory of Biomedical Optical Imaging Technology, Shenzhen Institute of Advanced Technology, Chinese Academy of Sciences, Shenzhen, 518055 China; 2 PKU-IDG–McGovern Institute for Brain Research, Beijing, 100871 China; 3 State Key Laboratory of Brain and Cognitive Science, Institute of Biophysics, Chinese Academy of Sciences, Beijing, 100101 China; 4 Institute of Neuroscience, State Key Laboratory of Neuroscience, CAS Center for Excellence in Brain Science and Intelligence Technology, Chinese Academy of Sciences, Shanghai, 200031 China; 5 Molecular Imaging Center, Guangdong Provincial Key Laboratory of Biomedical Imaging, The Fifth Affiliated Hospital, Sun Yat-sen University, Zhuhai, 519000 China; 6 Department of Oral Emergency and General Dentistry, Guangzhou Key Laboratory of Basic and Applied Research of Oral Regenerative Medicine, Guangdong Engineering Research Center of Oral Restoration and Reconstruction, Affiliated Stomatology Hospital of Guangzhou Medical University, Guangzhou, 510182 Guangdong China; 7 State Key Laboratory of Neuroscience, Institute of Neuroscience, Shanghai Institutes for Biological Sciences, Center for Excellence in Brain Science and Intelligence Technology, Chinese Academy of Sciences, Shanghai, 200031 China; 8 Department of Biochemistry and Molecular Biophysics, Kansas State University, Manhattan, 66506 KS USA; 9 Institute of Biomedicine and Biotechnology, Shenzhen Institute of Advanced Technology, Chinese Academy of Sciences, Shenzhen, 518055 China; 10 Brain Cognition and Brain Disease Institute, Shenzhen Institute of Advanced Technology, Chinese Academy of Sciences, Shenzhen, 518055 China; 11 Key Laboratory of Molecular Biophysics of the Ministry of Education, College of Life Science and Technology, Huazhong University of Science and Technology, Wuhan, 430074 China; 12 Vollum Institute, Oregon Health and Science University, Portland, 97239 OR USA; 13 Department of Experimental Medicine, The Fifth Affiliated Hospital, Sun Yat-sen University, Zhuhai, 519000 China; 14 Shenzhen-Hong Kong Institute of Brain Science, and Shenzhen Institute of Synthetic Biology, Shenzhen, 518055 China; 15 CAS Key Laboratory of Health Informatics, Shenzhen Institute of Advanced Technology, Chinese Academy of Sciences, Shenzhen, 518055 China

**Keywords:** Neuroscience, Fluorescent proteins

## Abstract

cAMP is a key second messenger that regulates diverse cellular functions including neural plasticity. However, the spatiotemporal dynamics of intracellular cAMP in intact organisms are largely unknown due to low sensitivity and/or brightness of current genetically encoded fluorescent cAMP indicators. Here, we report the development of the new circularly permuted GFP (cpGFP)-based cAMP indicator G-Flamp1, which exhibits a large fluorescence increase (a maximum Δ*F*/*F*_0_ of 1100% in HEK293T cells), decent brightness, appropriate affinity (a *K*_d_ of 2.17 μM) and fast response kinetics (an association and dissociation half-time of 0.20 and 0.087 s, respectively). Furthermore, the crystal structure of the cAMP-bound G-Flamp1 reveals one linker connecting the cAMP-binding domain to cpGFP adopts a distorted β-strand conformation that may serve as a fluorescence modulation switch. We demonstrate that G-Flamp1 enables sensitive monitoring of endogenous cAMP signals in brain regions that are implicated in learning and motor control in living organisms such as fruit flies and mice.

## Abstract (web summary)

The second messenger cAMP modulates a wide range of important biological processes. Here, the authors developed a fluorescent indicator termed G-Flamp1, which can accurately report levels of cAMP in living cells.

## Introduction

Cyclic adenosine 3′,5′-monophosphate (cAMP), which is produced from adenosine triphosphate (ATP) by adenylyl cyclase (AC), acts as a key second messenger downstream of many cell surface receptors, especially G-protein-coupled receptors (GPCRs)^1^. cAMP plays critical roles in regulating numerous cellular physiological processes, including neuronal plasticity and innate and adaptive immune cell activities, through its effector proteins such as protein kinase A (PKA), exchange protein directly activated by cAMP (EPAC), and cyclic nucleotide-activated ion channels (CNG and HCN channels)^2^. A growing body of evidence has shown that cAMP is precisely controlled in space and time in living cells and its abnormal dynamics are associated with many diseases^3^. However, it is largely unclear how cAMP signaling is regulated under physiological and pathological conditions in vivo^3–5^.

Genetically encoded fluorescent indicators (GEFIs) with advanced optical imaging have emerged as a powerful tool for real-time monitoring the spatiotemporal dynamics of signaling molecules including calcium in intact model organisms^6^. Current GEFIs for cAMP were developed based on two strategies: fluorescence resonance energy transfer (FRET) between two fluorescent proteins (FPs) or circular permutation/splitting of a single FP^7–9^. The latter is much more sensitive and, because they only require a single-color channel, can be more easily used together with other spectrally compatible sensors and actuators^10^. So far, a few single-FP cAMP sensors (Flamindo2, cAMPr, Pink Flamindo and R-FlincA) based on different mammalian cyclic nucleotide-binding domains (CNBDs) and green/red FPs have been created^11–14^. However, they exhibit small fluorescence changes (|Δ*F*/*F*_0_| < 150%) and most are dim in mammalian cells at 37 °C (Supplementary Fig. 1a–c). Thus, it is highly desirable to develop new high-performance (high brightness, high sensitivity and fast response kinetics) single-FP cAMP sensors that can decipher complex cAMP signals in vivo.

To address these problems, we engineered a highly responsive circularly permuted GFP (cpGFP)-based cAMP sensor named G-Flamp1 (green fluorescent cAMP indicator 1) by inserting cpGFP into the CNBD of the bacterial *Mloti*K1 channel (mlCNBD), followed by extensive screening. G-Flamp1 exhibits a maximum Δ*F*/*F*_0_ of 1100% in HEK293T cells at 37 °C, which is 9–47 times greater than existing single-FP cAMP sensors. Furthermore, we resolved the crystal structure of cAMP-bound G-Flamp1 and found a long distorted β-strand connecting mlCNBD and cpGFP, which is unseen in other single-FP sensors and could critically modulate sensor fluorescence. Finally, we successfully monitored cAMP signals with G-Flamp1 during learning and motor control in fruit flies and mice.

## Results

### Development of G-Flamp1

**Fig. 1:**
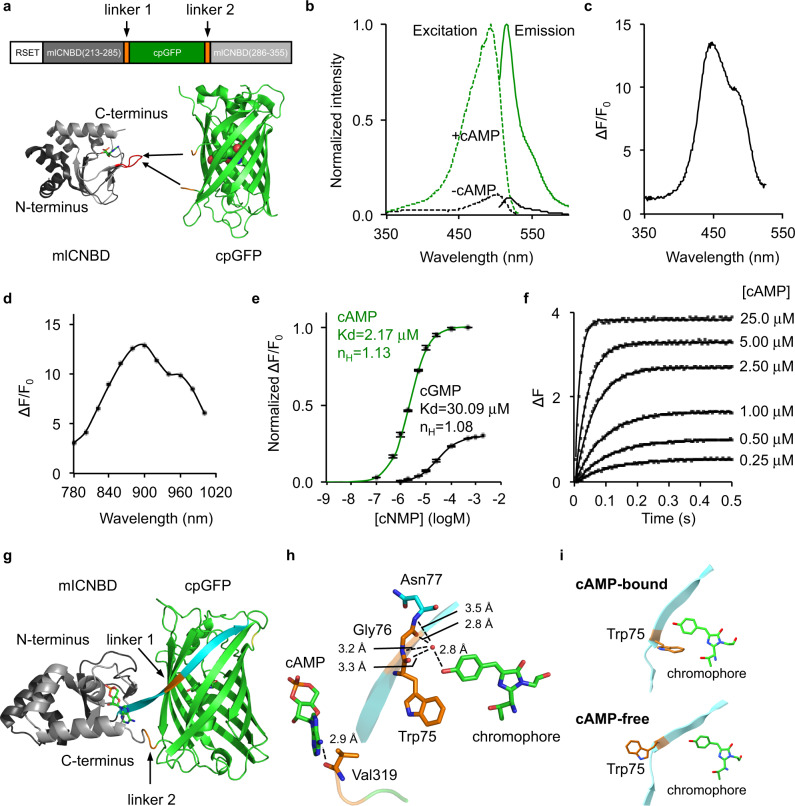
Development and in vitro characterization of G-Flamp1 indicator. **a** Schematic of G-Flamp sensors. cpGFP with two flanking linkers (two amino acids per linker) is inserted into mlCNBD (Gly213-Ala355, Genbank accession number: BA000012.4). The N-terminal peptide (RSET) including a 6× His tag is from the bacterial expression vector pNCS. The X-ray crystal structures of cAMP-bound mlCNBD (PDB: 1VP6) and cpGFP (PDB: 3WLD) are shown as cartoon with cAMP and chromophore of cpGFP shown as stick and sphere, respectively. The loop bearing the insertion site in G-Flamp1 is marked in red. **b** Excitation and emission spectra of cAMP-free and cAMP-bound G-Flamp1 sensors in HEPES buffer (pH 7.15). **c** Excitation wavelength-dependent Δ*F*/F_0_ of G-Flamp1 under one-photon excitation. **d** Excitation wavelength-dependent Δ*F*/*F*_0_ of G-Flamp1 under two-photon excitation. **e** Binding titration curves of G-Flamp1 to cAMP or cGMP in HEPES buffer (pH 7.15). The data were fitted by a sigmoidal binding function to extract the dissociation constant *K*_d_ and Hill coefficient *n*_H_. Data are presented as mean ± standard error of mean (SEM) from three independent experiments. **f** Binding kinetics of G-Flamp1 to cAMP measured using the stopped-flow technique in HEPES buffer (pH 7.15). Each curve corresponds to a different concentration of cAMP, i.e., from bottom to top: 0.25, 0.5, 1, 2.5, 5, and 25 μM. The data were fitted by a single-exponential function. **g** Cartoon representation of crystal structure of cAMP-bound G-Flamp1 (PDB: 6M63). The N- and C-terminal fragments of mlCNBD are shown in dark and light gray, respectively. cpGFP is in green and both linkers are in orange. The long β-strand possessing linker 1 is in cyan. **h** Chromophore and cAMP are in close proximity with linker 1 and linker 2, respectively. **i** Zoom-in view of Trp75 and the chromophore of simulated cAMP-bound and cAMP-free structures at conformations associated with the global minimum energy. Source data are provided as a Source Data file.

To develop a high-performance genetically encoded cAMP indicator (GEAI), we chose mlCNBD as a starting point (Fig. 1a), which was previously used to create the mlCNBD-based FRET indicator^15,16^. Unlike mammalian CNBDs, the bacterial mlCNBD likely does not interact with endogenous eukaryotic proteins and thus would not interfere with signaling pathways in mammalian cells^8^. Furthermore, mlCNBD exhibits high binding affinity and specificity for cAMP because the dissociation constants (*K*_d_) for mlCNBD-cAMP and mlCNBD-cGMP complexes are 68 and 499 nM, respectively. In addition, it has fast response kinetics with an association half-time (*t*_on_) of 27 ms under 1 μM cAMP and dissociation half-time (*t*_off_) of 74 ms^16^. Lastly, although mlCNBD is a homolog of mammalian CNBDs with a similar fold, its amino acid sequence and cAMP-binding pocket is significantly different from those of mammalian CNBDs (Supplementary Fig. 2), raising the possibility that cAMP sensors with a different response profile (brightness, fluorescence change, affinity, and kinetics) can be engineered.

To determine the optimal insertion site, we varied the position of cpGFP with original linkers from the calcium sensor GCaMP6f (LE-cpGFP-LP)^17^ in three loop regions of mlCNBD: the region ‘Gln237-Leu239’ undergoes a large conformation change from random coil to α-helix upon cAMP binding while the regions ‘Ala283-Val288’ and ‘Ala313-Val317’ remain random coils with small conformation change (numbering according to PDB 1VP6 of mlCNBD). A total of 11 sensors were tested using a bacterial lysate screening assay. One dim variant named G-Flamp0.1, in which cpGFP was inserted between Pro285 and Asn286 of mlCNBD, gave the largest signal change with a Δ*F*/*F*_0_ of −25.8% (Fig. 1a and Supplementary Fig. 3a–c). To improve the brightness of G-Flamp0.1, we examined several beneficial mutations from the well-folded GFP variants (Citrine and superfolder GFP)^18,19^ and generated G-Flamp0.2 with brightness increased by 330% (Supplementary Fig. 3d). To obtain a large Δ*F*/*F*_0_ sensor, both linkers connecting cpGFP and mlCNBD were randomized together. Of the 427 variants tested, one variant (G-Flamp0.5) with linkers ‘WG’ and ‘RV’ showed the largest fluorescence change with a Δ*F*/*F*_0_ of 230% when excited at 488 nm (Supplementary Fig. 3e). Next, we performed random mutagenesis on G-Flamp0.5 using error-prone PCR and were able to identify a bright and highly responsive variant G-Flamp0.7 with a Δ*F*/*F*_0_ of 560%, which harbors P285N mutation in mlCNBD and D173G mutation in GFP (Supplementary Fig. 3f, g). Finally, to increase the selectivity for cAMP over cGMP (defined as the *K*_d_ ratio of cGMP/cAMP), the mutation S308V, which is in the cAMP-binding pocket, was introduced to weaken the binding between mlCNBD and cGMP^20^. The resultant sensor G-Flamp1 had a higher selectivity with a Δ*F*/*F*_0_ of 820% under excitation at 488 nm (Supplementary Fig. 3g and Supplementary Fig. 4).

### In vitro characterization of G-Flamp1 sensor

We first investigated the fluorescence and absorption properties of purified G-Flamp1. The cAMP-bound G-Flamp1 had excitation and emission peaks at 490 and 510 nm, respectively, which were similar to those of mEGFP. The excitation and emission peaks of cAMP-free G-Flamp1 were redder than those of cAMP-bound G-Flamp1 by 10 nm and 3 nm, respectively (Fig. 1b and Supplementary Fig. 5a, b), suggesting different chromophore environments in cAMP-bound and cAMP-free G-Flamp1. According to these fluorescence spectra, the calculated fluorescence change peaked at 450 nm with a maximum Δ*F*/*F*_0_ of 1300% (Fig. 1c). Absorbance spectra revealed that both cAMP-bound and cAMP-free G-Flamp1 displayed two peaks with maxima at 400 and 490 nm (cAMP-bound G-Flamp1) or 500 nm (cAMP-free G-Flamp1) (Supplementary Fig. 5c), which correspond to protonated (dark state) and deprotonated (bright state) chromophores, respectively^21^. Moreover, the deprotonated form of cAMP-bound G-Flamp1 significantly increased, making it much brighter than deprotonated cAMP-free G-Flamp1. Under two-photon illumination, cAMP-bound G-Flamp1 had a similar excitation spectrum to mEGFP with a peak at around 920 nm (Supplementary Fig. 5d) and a maximum Δ*F*/*F*_0_ of 1300% at around 900 nm (Fig. 1d).

Compared to cAMP-free G-Flamp1, cAMP-bound G-Flamp1 exhibited a six-fold greater extinction coefficient (EC) (25280 mM^−1^cm^−1^ versus 4374 mM^−1^cm^−1^ at 488 nm) and similar quantum yield (QY) (0.322 versus 0.323) (Supplementary Table 1). The fluorescence change (6-fold) based on the measured EC and QY values is smaller than the Δ*F*/*F*_0_ of ∼8.5 under 488 nm excitation (Fig. 1c), which is also observed in recent GCaMP sensors (Supplementary Table 2)^22^. Like other single-FP probes, the fluorescence intensity of G-Flamp1 was sensitive to pH, with pKa values of 8.27 and 6.95 for cAMP-free and cAMP-bound G-Flamp1, respectively (Supplementary Fig. 6a). Moreover, the calculated Δ*F*/*F*_0_ peaked at pH 6.5 with a value of 1640% and remained high at pH 7.0 with a value of 1440% excited at 450 nm (Supplementary Fig. 6b), indicating that G-Flamp1 would be highly responsive in mammalian cells where the physiological pH is maintained between 6.8 and 7.3^23^.

The concentration-response curves showed that *K*_d_ values of G-Flamp1 for cAMP and cGMP were 2.17 and 30.09 μM, respectively (Fig. 1e), leading to a 13-fold higher selectivity for cAMP over cGMP, which is similar to other widely used cAMP probes (Supplementary Table 3)^14^. In addition, the maximum Δ*F*/*F*_0_ of G-Flamp1 induced by cGMP was only 30% of that induced by cAMP. Since the *K*_d_ value for G-Flamp1-cAMP complex is close to the resting cAMP concentration of 0.1–1 μM^24,25^, G-Flamp1 should detect cAMP changes under physiological stimulation conditions. To measure response kinetics, we applied the stopped-flow technique on purified G-Flamp1 and fitted data with a mono-exponential function. The apparent association (*k*_on_) and dissociation (*k*_off_) rate constants were 3.48 μM^−1^s^−1^ and 7.9 s^−1^, resulting in a *t*_on_ of 0.20 s under 1 μM cAMP and *t*_off_ of 0.087 s, respectively (Fig. 1f). Compared to the previously reported mlCNBD-based FRET indicator, G-Flamp1 has a lower affinity (*K*_d_ of 2.17 μM versus 0.066 µM) and a slower response speed (*k*_on_ of 3.48 μM^−1^s^−1^ versus 25 μM^−1^s^−1^). Regardless, our results indicate that G-Flamp1 can faithfully report cAMP dynamics with sub-second temporal resolution.

### Crystal structure of cAMP-bound G-Flamp1

To understand the molecular mechanism of large fluorescence change in the G-Flamp1 indicator, we determined the X-ray crystal structure of cAMP-bound G-Flamp1 without RSET tag at pH 8.0 to a 2.2 Å resolution (Fig. 1g). The statistics of data collection and structure refinement were summarized in Supplementary Table 4. Overall, all residues in G-Flamp1 showed good electron density except for N-terminal nine residues (MGFYQEVRR), C-terminal six residues (GAAASA) and a flexible linker (GGTGGS) within cpGFP. Two G-Flamp1 molecules were arranged as a dimer in one asymmetric unit of G-Flamp1 crystal and were structurally similar with an r.m.s.d. of Cα atoms of 0.149 Å. However, this homodimer was not biologically relevant and is likely caused by crystallographic packing because its dimerization interface is mediated by β-barrel ends of cpGFP rather than the previously described β-barrel wall^26^. Consistent with this, gel filtration analysis showed that G-Flamp1 at a high concentration of 0.7 mM was a monomer (Supplementary Fig. 7).

The linkers connecting sensing domain and circularly permuted FP (cpFP) are the main determinant of the dynamic range of single-FP sensors^9^. The crystal structure of cAMP-bound G-Flamp1 reveals that the first linker Trp75/Gly76 and the second linker Arg318/Val319 (numbering according to PDB 6M63 of G-Flamp1, Supplementary Fig. 4c), along with their flanking amino acids from mlCNBD and cpGFP, adopt a highly twisted β-strand and random coil conformation, respectively (Fig. 1g and Supplementary Fig. 8), which is unique because both linkers in other single-FP sensors with crystal structures available fold as random coil or α-helix segments (Supplementary Fig. 8)^27–29^. In G-Flamp1, linker 1 and linker 2 are in close proximity with chromophore and cAMP, respectively (Fig. 1h), suggesting the former primarily contributes to fluorescence change. Moreover, since the mlCNBD domain is far away from the chromophore, we reasoned that a self-contained fluorescence modulation mechanism, in which residues from linkers and/or FP (e.g., the red calcium sensor K-GECO1) rather than sensing domain (e.g., the green calcium sensor GCaMP3) interact with the deprotonated chromophore (Supplementary Fig. 9)^27^, may exist in G-Flamp1.

A close examination of linker 1 revealed that the Trp75 stabilizes the phenolic group of the chromophore in two ways. First, the backbone CO or NH groups of the tripeptide Trp75-Gly76-Asn77 indirectly interact with the phenolic oxygen of the chromophore, via a water molecule, to form a hydrogen-bonding network. This is expected to stabilize the anionic state of the chromophore in the cAMP-bound state. Moreover, such a hydrogen-bonding network reduces chromophore motions, which is expected to improve the quantum yield. Second, the bulky side chain of Trp75 protects the chromophore from solvent quenching^30^. Thus, we reasoned that a movement of Trp75 would make the chromophore unstable and dim. Consistent with this, metadynamics molecular dynamics simulations of cAMP-free and cAMP-bound G-Flamp1 showed that the side chain of Trp75 rotates away from the chromophore at conformation associated with the global minimum energy in the cAMP-free form (Fig. 1i and Supplementary Fig. 10). Subsequent saturation mutagenesis on position 75 demonstrated that all G-Flamp1 variants had reduced fluorescence changes with a Δ*F*/*F*_0_ of 0−232% (Supplementary Fig. 11), further confirming the critical role of Trp75 in tuning fluorescence change of G-Flamp1 in a self-contained manner. This makes the cpGFP as well as linkers in G-Flamp1 a useful scaffold to be combined with other sensing domains for engineering of new single-FP sensors. However, to verify these assumptions, a crystal structure of cAMP-free G-Flamp1 needs to be resolved and compared to that of cAMP-bound G-Flamp1.

### Performance of G-Flamp1 in mammalian cells

**Fig. 2:**
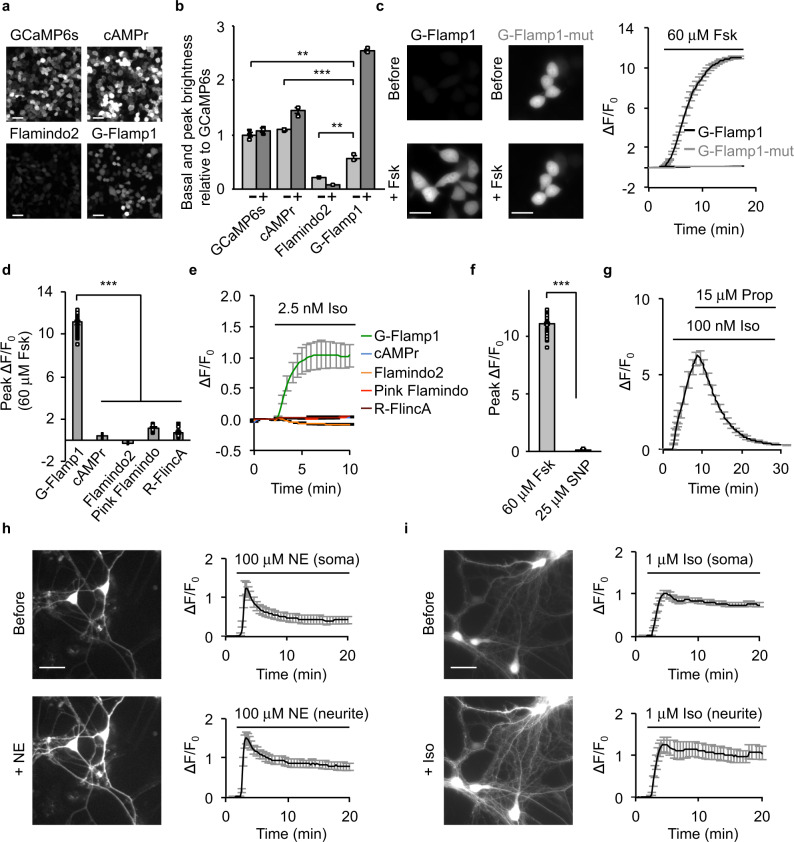
Characterization of G-Flamp1 in mammalian cells. **a** Representative fluorescence images of the sensors in resting HEK293T cells from three independent experiments. Scale bars: 50 μm. **b** Basal and peak brightness of the sensors in HEK293T cells (excited at 488 nm). *n* = 3 cultures for each indicator. *P* = 0.0033, 8.6 × 10^−4^ and 0.0069 between G-Flamp1 and GCaMP6s, cAMPr and Flamindo2, respectively. The minus and plus signs denote without and with 15 min Forskolin (Fsk) treatment, respectively. **c** Representative fluorescence images (left) and traces of Δ*F*/*F*_0_ (right) excited at 450 nm in response to 60 μM Fsk in HEK293T cells. *n* = 34 (G-Flamp1) and 22 (G-Flamp1-mut) cells from 3 independent experiments. Scale bars: 20 μm. **d** Peak ΔF/F_0_ in HEK293T cells. *n* = 34 (G-Flamp1), 33 (cAMPr), 42 (Flamindo2), 34 (Pink Flamindo) and 18 (R-FlincA) cells from 3 independent experiments. *P* = 3.5 × 10^−38^, 2.8 × 10^−38^, 1.4 × 10^−41^, and 1.6 × 10^−46^ between G-Flamp1 and cAMPr, Flamindo2, Pink Flamindo and R-FlincA, respectively. **e** Representative traces of Δ*F*/*F*_0_ in response to 2.5 nM Iso in HEK293T cells. *n* = 30 (G-Flamp1), 28 (cAMPr), 27 (Flamindo2), 27 (Pink Flamindo) and 14 (R-FlincA) cells from three independent experiments. **f** Peak Δ*F*/*F*_0_ of G-Flamp1 in response to 60 μM Fsk or 25 μM SNP in HEK293T cells. *n* = 34 (Fsk) and 15 (SNP) cells from three independent experiments. *P* = 4.3 × 10^−38^. **g** Representative traces of Δ*F*/*F*_0_ in response to 100 nM Iso followed by 15 μM Prop in HEK293T cells. *n* = 17 cells from 3 cultures. Representative fluorescence images (left) and traces of Δ*F*/*F*_0_ in response to 100 μM NE (**h**) or 1 μM Iso (**i**) in cortical neurons. *n* = 10 (soma) and 9 (neurite) regions of interest (ROIs) of 10 neurons from 3 cultures in **h** and *n* = 28 (soma) and 14 (neurite) ROIs of 28 neurons from 3 cultures in **i**. Scale bars: 20 μm.Data are presented as mean ± SEM in **b**, **c** (right), **d**–**h** (right) and **i** (right). Two-tailed Student’s *t*-tests were performed for above statistical analysis. ***P < 0.001 and **P < 0.05. Source data are provided as a Source Data file.

We first examined the cellular localization and brightness of G-Flamp1 in HEK293T cells. G-Flamp1, like Flamindo2 and Pink Flamindo, was evenly distributed in cytoplasm and nucleus (Fig. 2a and Supplementary Fig. 1b. The detailed imaging conditions throughout the paper are summarized in Supplementary Table 5). In contrast, cAMPr and R-FlincA were found to localize mainly in the cytosol (Fig. 2a and Supplementary Fig. 1b), with the latter forming puncta 48 h post transfection (Supplementary Fig. 1d) and thus likely being toxic to mammalian cells^27^. Under one-photon (488 nm) illumination, the basal fluorescence intensities of G-Flamp1, cAMPr and Flamindo2 were 57, 109, and 21% of that of GCaMP6s^17^, respectively (Fig. 2a, b). At 450 nm, which gives the largest Δ*F*/*F*_0_, the basal brightness is reduced by 66% and ∼19% of that of GCaMP6s taking the excitation efficiencies at 450 and 488 nm into account (Fig. 1b). Again, under two-photon (920 nm) illumination, G-Flamp1 was brighter than Flamindo2 but dimmer than cAMPr (74% versus 38% of Flamindo2 and 165% of cAMPr) in the resting state (Supplementary Fig. 12a).

Next we evaluated the cytotoxicity and interference with cAMP signaling of G-Flamp1 at a medium expression level. HEK293T cells stably expressing G-Flamp1 proliferated similarly to untransfected cells (Supplementary Fig. 13a), suggesting low cytotoxicity of G-Flamp1. To assess G-Flamp1’s buffering effect, we investigated the phosphorylation of cAMP response element binding protein (CREB) at Ser133, a key molecular event downstream of cAMP-PKA^31^. Both G-Flamp1-expressing HEK293T and control cells showed similar basal levels and increases of phospho-S133 of CREB before and after β-adrenergic receptor (β-AR) agonist isoproterenol (Iso) stimulation with low concentrations (10 nM or 100 nM), respectively (Supplementary Fig. 13b). Taken together, these results indicate that G-Flamp1 expression had no obvious effects on endogenous signaling.

We further determined the fluorescence change and sensitivity of G-Flamp1. Forskolin (Fsk), a potent activator of transmembrane AC^32^, was used to induce a high level of cAMP to assess the maximum fluorescence change. Under 450 nm illumination, G-Flamp1 expressed in HEK293T cells exhibited a maximum Δ*F*/*F*_0_ of 1100% in response to 60 μM Fsk, which was 9–47 times larger than those of other cAMP probes (Fig. 2c, d and Supplementary Fig. 1b). G-Flamp1 also showed large fluorescence increases with a maximum Δ*F*/*F*_0_ of 340 and 820% in HeLa and CHO cells, respectively (Supplementary Fig. 14). To rule out possible unspecific responses, we generated a cAMP-insensitive indicator G-Flamp1-mut by introducing the R307E mutation into mlCNBD of G-Flamp1 (Supplementary Fig. 15)^20^. As expected, G-Flamp1-mut showed no detectable signal change in living cells (Fig. 2c). Notably, the 480 nm excitation gave a much smaller Δ*F*/*F*_0_ than 450 nm excitation (250% versus 1100%), leading to a smaller signal-to-noise ratio (SNR) (200 versus 361) (Supplementary Fig. 16). Therefore, 450 nm excitation was used for subsequent one-photon imaging experiments. To demonstrate the sensitivity of G-Flamp1, 2.5 nM Iso was exploited to produce a small amount of cAMP in HEK293T cells. G-Flamp1 exhibited an obvious fluorescence increase with a Δ*F*/*F*_0_ > 100% after 5 min stimulation while other sensors showed little signal changes (|Δ*F*/*F*_0_| < 10%) in our setup (Fig. 2e). Under two-photon excitation (920 nm), G-Flamp1 exhibited a maximum Δ*F*/*F*_0_ of 1240%, which is much larger than those of Flamindo2 and cAMPr (−79 and 72%, respectively) (Supplementary Fig. 12b, c). Meanwhile, G-Flamp1 had a 250-fold higher signal-to-noise ratio compared with Flamindo2 and cAMPr (Supplementary Fig. 12d). In addition, G-Flamp1 exhibited a small fluorescence lifetime decrease from 2.169 to 2.069 ns upon 60 μM Fsk treatment (Supplementary Fig. 17).

Then we explored the specificity and reversibility of G-Flamp1 in HEK293T cells. Cyclic guanosine monophosphate (cGMP), which is synthesized from guanosine triphosphate (GTP) by guanylyl cyclase in mammalian cells, has been shown to bind cAMP-sensing domains with weaker affinity^14,33^. To examine the response of G-Flamp1 to cGMP, the sodium nitroprusside (SNP), a nitric oxide (NO) donor that activates soluble guanylyl cyclase, was utilized to induce a large amount of cGMP in living cells. When HEK293T cells were treated with 25 μM SNP, the low-affinity (*K*_d_ ∼1.09 μM) cGMP sensor Green cGull^34^ showed a maximum Δ*F*/*F*_0_ of 210% while G-Flamp1 showed no detectable signal change (Fig. 2f and Supplementary Fig. 18), indicating the high specificity of G-Flamp1 towards cAMP over cGMP. Regarding reversibility, HEK293T cells expressing G-Flamp1 exhibited increased fluorescence upon 100 nM Iso treatment and then returned to basal level after addition of 15 μM β-AR antagonist propranolol (Prop) (Fig. 2g).

Besides cell lines, primary cortical neurons were also utilized to examine cellular localization and fluorescence change of G-Flamp1. Again, G-Flamp1 was evenly distributed in neuronal soma and neurites. Upon application of 100 μM AR agonist norepinephrine (NE) or 1 μM Iso, a Δ*F*/*F*_0_ of ∼100–150% was observed in both soma and neurites (Fig. 2h, i). Upon 60 μM Fsk treatment, G-Flamp1 showed significant fluorescence increase with a Δ*F*/*F*_0_ of 500–700% in both soma and neurites (Supplementary Fig. 19). Taken together, G-Flamp1 shows low cytotoxicity, great distribution, decent brightness, large dynamic range and high sensitivity in cell lines and primary neurons at 37 °C.

### In vivo two-photon imaging of cAMP dynamics in zebrafish

To test whether G-Flamp1 can function in intact living organisms, we first utilized optically transparent zebrafish embryos under Fsk stimulation. We injected UAS:G-Flamp1(or G-Flamp1-mut)-T2A-NLS-mCherry (nuclear-localized mCherry) plasmid into the embryos of EF1α:Gal4 transgenic zebrafish at one-cell stage (Supplementary Fig. 20a). The expression of G-Flamp1 or G-Flamp1-mut sensor was confirmed by green fluorescence in cells of the developing central nervous system. Brain ventricular injection of 120 μM Fsk but not PBS elicited a robust fluorescence increase with a Δ*F*/*F*_0_ of 450% for G-Flamp1, whereas no signal changes were observed for G-Flamp1-mut (Supplementary Fig. 20b–d). These data indicate that G-Flamp1 sensor has high sensitivity for in vivo cAMP detection in zebrafish.

### In vivo two-photon imaging of cAMP dynamics in *Drosophila*

**Fig. 3:**
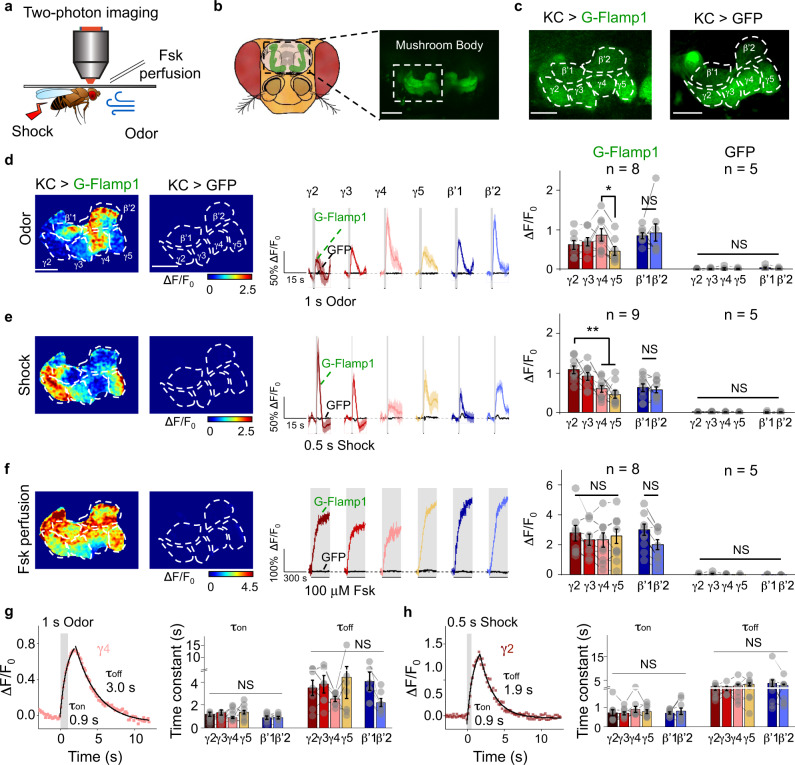
G-Flamp1 reports compartmental cAMP dynamics evoked by physiological stimuli in *Drosophila* through in vivo two-photon imaging. **a** Schematics of in vivo two-photon imaging setup in *Drosophila* with multiple stimuli. **b** Schematics and fluorescent images of *Drosophila* MB KCs. Scale bar: 50 μm. **c** Representative fluorescence images of *Drosophila* MB KCs expressing G-Flamp1 (left) or GFP (right) from 25 or 5 independent experiments. Scale bars: 25 μm. **d** Representative pseudo-color image (left), traces (center) and quantification (right) of Δ*F*/*F*_0_ to 1 s odor. Representative traces were 3-trial average from one fly. *n* = 8 and 5 for G-Flamp1 and GFP groups, respectively. Two-tailed Student’s *t*-tests were performed in d3. *P* = 0.21, 0.37 and 0.048 between γ4 and γ2, γ3 and γ5, respectively. Scale bars: 25 μm. **e** Similar to **d** except that 0.5 s electrical shock was applied to the fly. *n* = 9 and 5 for G-Flamp1 and GFP groups, respectively. Two-tailed Student’s *t*-tests were performed in e3. *P* = 0.368, 0.007 and 0.001 between γ2 and γ3, γ4 and γ5, respectively. **f** Similar to **d** except that 100 μM Fsk was perfused to the fly brain. *n* = 8 and 5 for G-Flamp1 and GFP groups, respectively. Two-tailed Student’s *t*-tests were performed in f3. *P* > 0.05 between γ2, γ3, γ4 and γ5. **g** Representative traces of Δ*F*/*F*_0_ of G-Flamp1 in γ4 evoked by 1 s odor. Data were fitted with single-exponential functions (left). Quantifications of *τ*_on_ and *τ*_off_ for different MB compartments were shown (right). *n* = 8 for each compartment. One-way ANOVA test was performed. NS not significant. **h** Similar to **g** except that 0.5 s electrical shock was applied to the fly. *n* = 9 for each compartment. One-way ANOVA test was performed. NS, not significant.Quantifications in **d**–**g** (right) and **h** (right) are shown as mean ± SEM. **P < 0.01, *P < 0.05 and NS not significant.Source data are provided as a Source Data file.

The importance of cAMP in associative learning, where it serves as a coincidence detector by integrating concurrent signal inputs from both conditioned and unconditioned stimuli, has been well documented across phyla^35,36^. In *Drosophila*, cAMP signaling in the mushroom body (MB) Kenyon cells (KCs) is indispensable for acquiring aversive memory, such as associating specific odor with punitive electrical shock^37,38^. To reveal cAMP dynamics in living organisms, we generated transgenic flies expressing G-Flamp1 in MB KCs and performed functional two-photon imaging in MB medial lobe (Fig. 3a–c). When the fly was exposed to either 1 s odor puff or subsequent 0.5 s electrical shock, we observed time-locked fluorescence responses with a Δ*F*/*F*_0_ of ∼100% (Fig. 3d, e). Compared with the MB β’ lobe that has similar responses among different compartments, the MB γ lobe exhibited compartmentally heterogeneous responses to specific stimuli, as the largest responses were observed in γ4 to odor and in γ2 to electrical shock. These compartmentalized signals were not due to the unequal expression level or saturation of the sensor, since 100 μM Fsk perfusion elicited a homogeneous Δ*F*/*F*_0_ of around 250% (Fig. 3f). G-Flamp1 specifically reported cAMP changes since the GFP alone expressed in KCs showed no significant response to 1 s odor, 0.5 s shock or 100 μM Fsk perfusion (Fig. 3d–f). Moreover, both the rise and decay time (*τ*_on_ and *τ*_off_) for cAMP changes evoked by odor or shock were similar in different compartments (Fig. 3g, h). The distinct compartmental cAMP signals in the MB during odorant or body shock delivery suggest the functional independence of different MB compartments in associative learning^39,40^, consistent with the heterogeneous MB dopamine (DA) signals^41,42^. Collectively, these results show that G-Flamp1 allows detection of physiologically relevant cAMP dynamics in *Drosophila* with high fidelity and good spatiotemporal resolution, and sheds light on the role of compartmentally separated cAMP signaling in the olfactory learning process.

### In vivo two-photon imaging of cAMP dynamics in mouse cortex

**Fig. 4:**
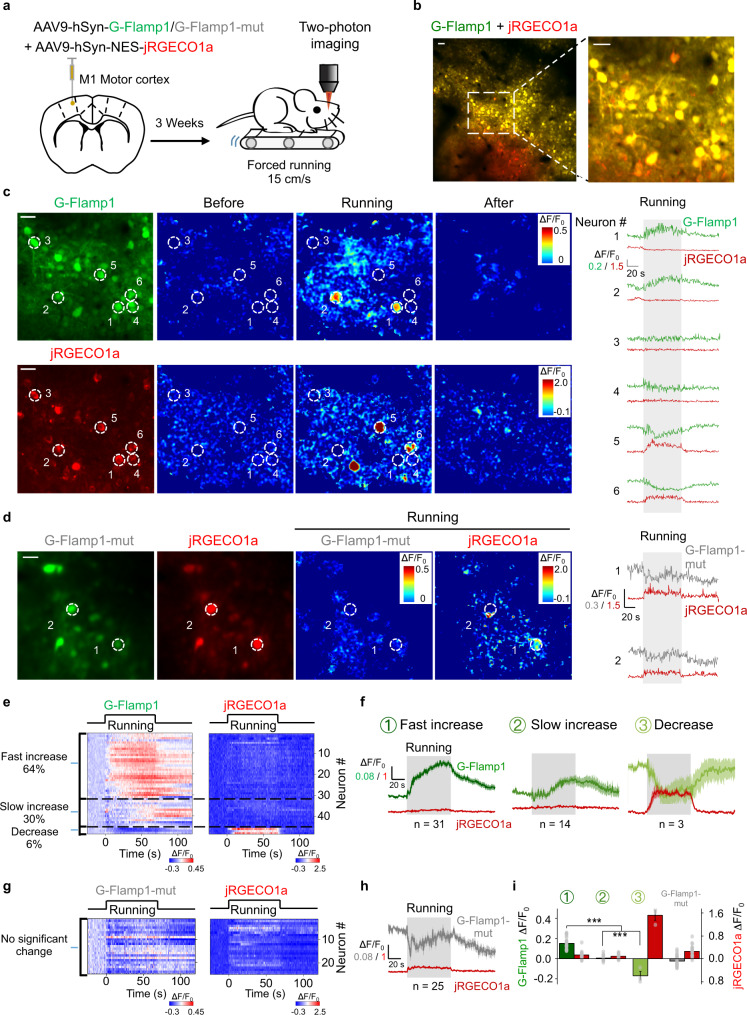
G-Flamp1 reveals forced running-induced cAMP signals of neurons in the mouse motor cortex through in vivo two-photon imaging. **a** Schematic diagram depicting the head-fixed mice on a treadmill together with two-photon imaging of the motor cortex co-expressing G-Flamp1 (or G-Flamp1-mut) and jRGECO1a. **b** Two-photon imaging of the mouse motor cortex co-expressing G-Flamp1 and jRGECO1a. The fluorescence of G-Flamp1 (green) and jRGECO1a (red) was merged and shown in yellow pseudo-color. Scale bars: 50 μm. **c** Representative images of probe expression in mice (left), the pseudo-color images (center) and the traces (right) of Δ*F*/*F*_0_ in response to forced running. White dashed circles with a diameter of 20 μm indicate selected ROIs covering soma for analysis. Scale bars: 30 μm. **d** Representative images of probe expression in mice (left), the pseudo-color images of Δ*F*/*F*_0_ (center) during the forced running phase and the traces of Δ*F*/*F*_0_ (right) in response to forced running. The white dashed circles with a diameter of 20 μm indicate selected ROIs covering the soma for analysis. Scale bar: 30 μm. **e** Heatmaps of G-Flamp1 and jRGECO1a responses during running task. Each row denotes a single cell’s response. *n* = 48 cells from three mice. **f** Averaged traces of Δ*F*/*F*_0_ for G-Flamp1 and jRGECO1a for neurons from three groups of different cAMP dynamics. *n* = 31, 14 and 3 cells for fast increase, slow increase and decrease groups, respectively. **g** Heatmaps of G-Flamp1-mut and jRGECO1a responses during running task. *n* = 25 cells from three mice. **h** Averaged traces of Δ*F*/*F*_0_ for G-Flamp1-mut and jRGECO1a during forced running process. **i** Quantification of the average Δ*F*/*F*_0_ during the first 30 s after the onset of forced running for G-Flamp1, G-Flamp1-mut and jRGECO1a in **e**, **g**. *n* = 31, 14, and 3 neurons from three mice for groups 1, 2 and 3, respectively. *n* = 25 from three mice for G-Flamp1-mut group. Two-tailed Student’s *t*-tests were performed. ***P < 0.001.Quantifications are shown as mean ± SEM in **f**, **h**, **i** with shaded regions or error bars indicating the SEM. Source data are provided as a Source Data file.

To demonstrate the utility of G-Flamp1 sensor to detect physiologically relevant cAMP dynamics in living animals, we performed head-fixed two-photon imaging in the motor cortex (M1) of awake mice during forced locomotion (Fig. 4a), which was reported to be associated with increased neuromodulator and PKA activities^43^. We co-expressed G-Flamp1 (or G-Flamp1-mut) and the red calcium sensor jRGECO1a in the neurons of motor cortex and imaged the layer 2/3 region (Fig. 4b). We observed running-induced, cell-specific, cAMP and calcium signals (Fig. 4c). Interestingly, neurons in M1 area could be further divided into three groups based on the cAMP dynamics: ∼64% neurons with fast increase of cAMP (higher average response during the first 30 s after the onset of forced running) and no significant change of calcium, ∼30% neurons with slow increase of cAMP and little change of calcium, and ∼6% neurons with decrease of cAMP and increase of calcium (Fig. 4c). The first two groups had no significant correlation while the third group showed a negative correlation between the cAMP and calcium signals by the Pearson correlation analysis (Supplementary Fig. 21). As a control, G-Flamp1-mut showed little fluorescence change (Fig. 4d). Distribution analysis and averaged traces of Δ*F*/*F*_0_ of G-Flamp1 and jRGECO1a further confirmed the heterogeneity of neuronal responses (Fig. 4e–i). Regarding the heterogeneous cAMP signals in the neurons, they may be elicited by different upstream neuromodulators in response to the forced running and/or differential expression of neuromodulatory receptor subtypes^44^. In addition, the inconsistency between the cAMP signals and calcium activities in the majority of neurons indicates that they are regulated in a relatively independent manner.

### In vivo fiber photometry recording of cAMP dynamics in mouse nucleus accumbens

**Fig. 5:**
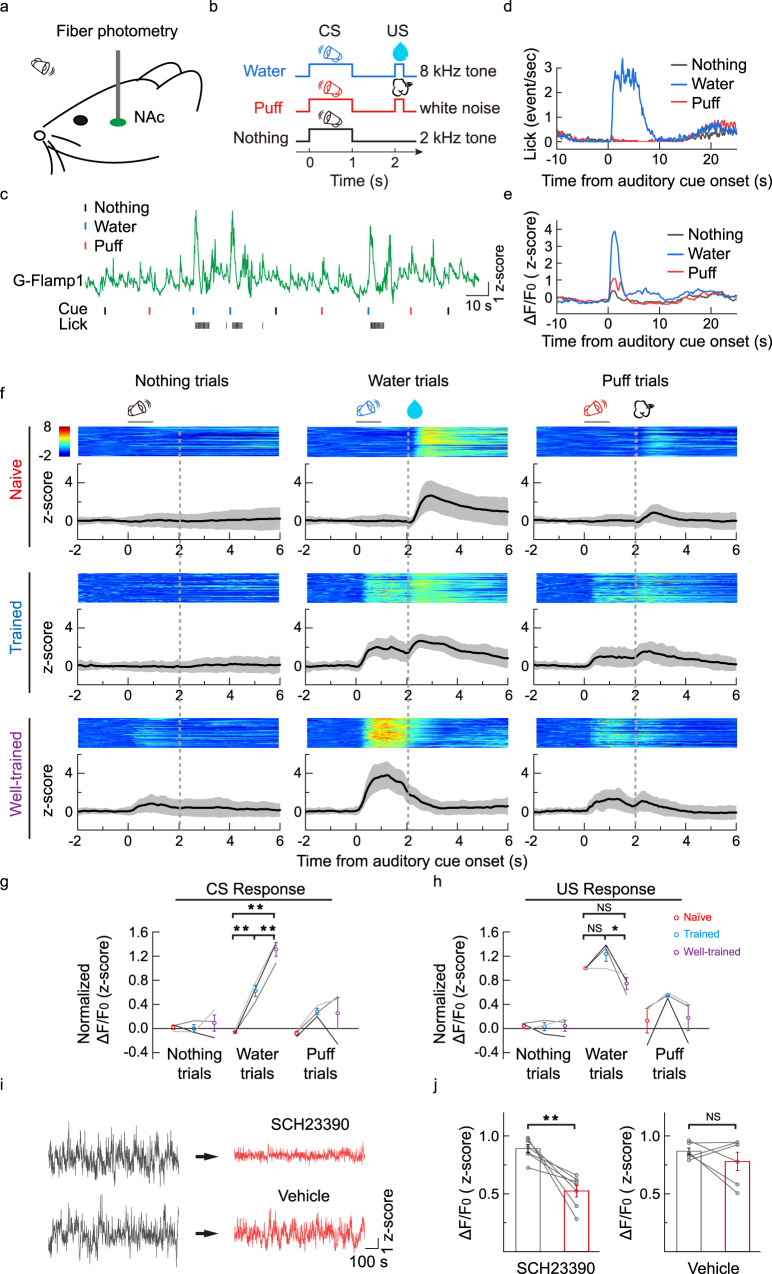
G-Flamp1 reports cAMP activities during an auditory Pavlovian conditioning task in the mouse NAc through in vivo fiber photometry. **a** Schematic for fiber photometry recording of G-Flamp1-expressing neurons from the NAc of a head-fixed mouse during an auditory Pavlovian conditioning task. **b** Schematic diagrams for the behavioral tasks. The mouse was trained to learn associations between three different auditory cues (conditioned stimulus, CS) and corresponding outcomes (unconditioned stimulus, US). **c** Exemplar trace of G-Flamp1 signal from a well-trained mouse encompassing nine sequential trials. The timings of cues (CS) and the lick responses (US) are indicated below. **d** Exemplar time-aligned lick responses in **c**. **e** Exemplar time-aligned G-Flamp1 signals in **c**. **f** Exemplar time-aligned pseudo-color images and averaged traces (mean shaded with ± standard deviation) from a mouse in naïve, trained and well-trained sessions. **g**, **h** Group analysis of the normalized peak Z scores of cAMP signals to CS and US in different sessions. Each trace (coded with specific gray value) represents data from one animal (*n* = 3 mice). Values with error bars indicate mean ± SEM. Two-tailed Post hoc Tukey’s tests were performed. Water trial CS responses: *P* = 0.00312 between naive and trained, *P* = 6.92772 × 10^−5^ between naive and well-trained, *P* = 0.00312 between trained and well-trained. Water trial US responses: *P* = 0.23198 between naïve and trained, *P* = 0.19808 between naive and well-trained, *P* = 0.02021 between trained and well-trained. **i** Exemplar recording of G-Flamp1 signals in NAc before and after injection (i.p.) of D1R antagonist SCH23390 or vehicle. **j** Quantification of G-Flamp1 signals before and after SCH23390 (*n* = 7 recordings from 3 mice, *P* = 0.0038) or vehicle (*n* = 6 recordings from 3 mice, *P* = 0.34) injection. Data are presented as mean ± SEM. Two-tailed Student’s *t*-tests were performed in **j**. **P < 0.01, *P < 0.05 and NS not significant. Source data are provided as a Source Data file.

To test the ability of G-Flamp1 sensor to report cAMP dynamics in deep brain regions, we measured cAMP levels in the nucleus accumbens (NAc) using fiber photometry in mice performing a classical conditioning task. The NAc was chosen because it is recently reported that PKA, a downstream molecule in the cAMP signaling pathway, plays a critical role in dopamine-guided reinforcement learning behavior^45^. We first injected an adeno-associated virus (AAV) expressing G-Flamp1 into the NAc and measured fluorescence signals using fiber photometry while the mice were trained to perform the conditioning task (Fig. 5a). In the task, the mice were trained to learn the associations between three auditory cues (conditioned stimulus, CS) and respective outcomes (unconditioned stimulus, US) (Fig. 5b; 8 kHz pure tone → water; white noise → brief air puff to the animal’s face; 2 kHz pure tone → nothing). Well-trained mice had a high licking rate selectively to the water-predictive sound, and the G-Flamp1 signal showed a large increase immediately after the onset of the water-predictive sound, while responses to the other two sounds were much smaller (Fig. 5c–e).

Interestingly, the G-Flamp1 signal in the water trials exhibits characteristic dynamics during the learning process: in naïve mice, there was a notable signal increase to water delivery; throughout the training, the magnitude of the water-evoked response decreased, while a response to the reward-predictive sound gradually increased (Fig. 5f–h). This dynamic change mimics the dopamine signal during classical conditioning^42,46^, suggesting that the increase in cAMP in the NAc is mainly driven by dopamine release. To confirm this, we thus blocked the dopamine D1 receptor using SCH23390 (i.p.) and observed a significantly reduced cAMP signal (Fig. 5i, j). The distinct cAMP activities in response to different stimuli and its dynamic change at different stages of the task demonstrate a highly specific and adaptive control of intracellular messengers in the changing external environment. Together, these results demonstrate that the G-Flamp1 sensor has a high signal-to-noise ratio and high temporal resolution to report the dynamic changes of cAMP in behaving mice.

## Discussion

In this study, we described G-Flamp1, a high-performance GEAI engineered by inserting cpGFP into the bacterial mlCNBD. G-Flamp1 exhibits a maximum Δ*F*/*F*_0_ of ∼1100% in living cells under both one-photon and two-photon excitation, thus being the most responsive GEAI. We also demonstrated the utility of G-Flamp1 in reporting cAMP dynamics in various model organisms with optical imaging and fiber photometry methods. To the best of our knowledge, this is the first time to demonstrate the cAMP activity in cortical neurons with a single-cell resolution in behaving animals and the dynamic change of cAMP level throughout associative learning in the mammalian brain, respectively. Given the high sensitivity and direct readout, G-Flamp1 would be useful for screening drugs targeting cAMP signaling pathways using high-content screening assays.

Our in vivo two-photon imaging experiments in mouse cortex showed that G-Flamp1 is able to detect bidirectional cAMP changes with single-neuron resolution (Fig. 4). Given that multiple neuromodulators can be released in the motor cortex^43^, different downstream signaling processes are expected to be induced in cortex neurons, which might partially explain the discrepancy between cAMP signal and calcium activity in our results (Fig. 4f). Further studies are needed to dissect out the underlying regulation mechanisms and potential functions. Nevertheless, together with other spectrally compatible sensors, G-Flamp1 will be a useful tool for investigating signal transduction networks in behaving animals.

In NAc, dopamine release can cause opposite dynamics to cAMP signaling through D1 or D2 receptors, which are expressed on two types of medium spiny neurons (MSNs; D1-MSNs and D2-MSNs)^47^. Thus, the different changes in the cAMP signals evoked by rewarding (water) or aversive (puff) stimuli may originate from D1- or D2-MSNs, respectively. To confirm this, further fiber photometry recording and/or single-cell-resolution imaging studies with G-Flamp1 labeled D1- or D2-MSNs are required. Additionally, cAMP signals measured in NAc were attenuated but not abolished by D1 receptor blockade, likely due to a submaximal dose of D1 receptor antagonist used, because a larger dose is known to make the animals unresponsive in the task^48^.

Very recently, three genetically encoded cAMP indicators (single FP-based Pink Flamindo and cADDis, FRET-based cAMPFIRE-L) have been used for two-photon imaging of cAMP in behaving mice^44,49,50^, building up the relationship between cAMP signaling and animal behavior. Given its high performance, G-Flamp1 would be an alternative and better choice for in vivo cAMP imaging. Compared to GCaMPs, the potential capabilities of G-Flamp1 are only beginning to be realized and will be fully explored in the future. Combined with miniaturized microscopes^51^, G-Flamp1 would be able to visualize cAMP activity patterns in freely moving animals. Moreover, by utilizing G-Flamp1 along with biological models, some long-standing biological questions may be addressed. For example, it may be possible to understand how cAMP is regulated in drug addiction and stress-induced behaviors^52,53^.

Engineering and structural analysis of G-Flamp1 reveals two interesting findings. First, modest conformation changes of insertion sites in sensing domain can induce large fluorescence change of cpFP. Generally, insertion sites with large structural change are chosen to make large fluorescence change sensors^54^. However, the insertion site in G-Flamp1 is near the mouth of the cAMP-binding pocket and undergoes a small conformational change upon cAMP binding^20^. Second, linkers connecting sensing domain and cpFP can adopt a more rigid conformation. Although random coil and short α-helical turns are observed in single-FP sensors with crystal structures available^27–29^, the first linker along with its flanking sequences in cAMP-bound G-Flamp1 folds as a long β-strand.

Despite its high performance, G-Flamp1 could be further improved for specific applications. It would be feasible to generate G-Flamp1 variants with improved properties through structure-guided mutagenesis. For example, G-Flamp1 variants with higher basal fluorescence may be useful to monitor cAMP activities in fine structures with high signal-to-background ratio. In addition, G-Flamp1 variants with higher affinity would enable more sensitive detection of subtle changes of cAMP at submicromolar concentration. Besides green G-Flamp1 and its variants, red/near-infrared and photoconvertible sensors using mlCNBD as a sensing domain could be developed to visualize cAMP changes in deep tissue and permanently mark cells with cAMP activities, respectively, which has been realized in calcium sensors^55–57^.

## Methods

This research complies with all relevant ethical regulations approved by Shenzhen Institute of Advanced Technology-CAS, Peking University, and Institute of Neuroscience-CAS.

### Chemicals and reagents

cAMP-Na (Cat. No. A6885) and cGMP-Na (Cat. No. G6129) were purchased from Sigma-Aldrich. cAMP (Cat. No. C107047), noradrenaline bitartrate monohydrate (N107258), isoproterenol hydrochloride (Cat. No. I129810), and propranolol (Cat. No. S133437) were purchased from Aladdin (Shanghai, China). Forskolin (Cat. No. S1612) and Enhanced Cell Counting Kit-8 (CCK-8) (Cat. No. C0041) were purchased from Beyotime Biotechnology (Shanghai, China). The CREB antibody 48H2 (Cat. No. 9197S) and phospho-CREB (Ser133) antibody 87G3 (Cat. No. 9198S) were purchased from Cell Signaling Technology, Inc.

### Plasmid construction

Plasmids were generated using the Infusion method (Takara Bio USA, Inc.). PCR fragments were amplified using PrimerStar (normal PCR or site-directed mutagenesis) or Taq (random mutagenesis) DNA polymerases. When needed, overlap PCR was exploited to generate the intact DNA fragment for Infusion. All PCR primers were purchased from Sangon Biotechnology Co., Ltd (Shanghai, China). Plasmids p2lox-cAMPr (Cat. No. 99143), pAAV.Syn.GCaMP6f.WPRE.SV40 (Cat. No. 100837), pAAV.CamKII.GCaMP6s.WPRE.SV40 (Cat. No. 107790) and pAAV.Syn.NES-jRGECO1a.WPRE.SV40 (Cat. No. 100854) were purchased from Addgene. The DNA sequences of Flamindo2, Pink Flamindo, mlCNBD and jRCaMP1b were synthesized by Genscript^11,13,16,58^. pcDNA4-R-FlincA was a gift from Dr. Kazuki Horikawa (Tokushima University). To express fluorescent proteins or sensors in bacterial or mammalian cells, cDNAs of FPs or sensors were subcloned into pNCS or pCAG vector^59^, respectively. To improve G-Flamp1’s stability in mammalian cells, its N-terminal arginine immediately after the initiator methionine was deleted^60^. cDNAs of G-Flamp1, G-Flamp1_opt_ and G-Flamp1-mut_opt_ (opt: mouse/human codon optimized) were subcloned into AAV vectors to make AAV2-CAG-G-Flamp1, AAV2-hSyn-G-Flamp1, and AAV2-hSyn-G-Flamp1-mut. pCAG-mEGFP and pCAG-mCherry were kept in our lab. All constructs were confirmed by DNA sequencing (Sangon Biotechnology Co., Ltd, Shanghai, China).

### Screening of cAMP sensors expressed in bacteria

Two mlCNBD fragments (Gly213-Pro285 and Asn286-Ala355) and cpGFP with linkers from GCaMP6f were amplified, overlapped and cloned into BamHI/EcoRI sites of pNCS vector with an N-terminal 6×His tag for protein purification. Site-directed and random mutagenesis were performed via overlap PCR and error-prone PCR, respectively. The DNA libraries were transformed into DH5α cells lacking adenylate cyclase gene *CyaA* (DH5α-*ΔCyaA*), which were generated by the phage λ Red recombination system^61^. After overnight incubation at 34 °C, colonies with different fluorescence intensities on the LB agar plates were screened by eye in a BlueView Transilluminator (Vernier) with the 400–500 nm excitation light and a yellow acrylic long-pass filter, or by fluorescence imaging in a home-made imaging system with 480/20 nm excitation and 520/20 nm emission filters. To quantitatively compare the brightness of selected variants, bacterial patches on the agar plates cultured overnight at 34 °C were: (1) imaged in the home-made system mentioned above and analyzed by ImageJ software (National Institutes of Health) (Supplementary Fig. 3d, f), or (2) collected in PBS and the OD_600_-normalized fluorescence intensities were measured using Tecan i-control 1.11 software of the Infinite M1000 fluorometer (Tecan) (Supplementary Fig. 11).

The fluorescence changes of cAMP sensors in response to cAMP were examined using the bacterial lysate. Briefly, selected bacterial colonies were patched on LB agar plate and grew at 25 °C for 3 days. The harvested bacterial cells were suspended in 1 mL of HEPES buffer (150 mM KCl and 50 mM HEPES-KOH, pH 7.15) and lysed by sonication followed by centrifugation. 120 μL of clear lysates were mixed with 2 μL of HEPES buffer or 2 μL of 30 mM cAMP or 2 μL of 30 mM cGMP and then the fluorescence were recorded with an Infinite M1000 PRO fluorometer (Tecan). The fluorescence change Δ*F*/*F*_0_ was calculated as (*F* – *F*_0_)/*F*_0_, where *F* and *F*_0_ are fluorescence intensities of sensors in the presence or absence of cAMP (or cGMP), respectively.

### Bacterial protein expression, purification, and in vitro characterization

DH5α-*ΔCyaA* cells were transformed with pNCS-FP or sensor and cultured overnight at 34 °C. The colonies were then patched on LB agar plates and cultured at room temperature for 3 days. The harvested bacterial cells were suspended in HEPES buffer and lysed by sonication. His-tagged recombination proteins were purified with cobalt-chelating affinity chromatography (Pierce) and desalted with HEPES buffer (pH 7.15) using the gel filtration column (Bio-Rad).

Quantum yields were determined using mEGFP as a standard (QY = 0.60). Extinction coefficients were determined according to the ‘base denatured chromophore’ method^59^. pH titrations were performed using a series of pH buffers ranging from 2 to 10.5 (50 mM Citrate-Tris-Glycine buffer. The desired pH was achieved by adding 2 M of sodium hydroxide or 2 M of hydrochloric acid)^59^. The fluorescence excited at 450 nm in different pH buffers was measured using an Infinite M1000 PRO fluorometer. The fluorescence intensities were plotted against the pH values and the pKa was determined by fitting the data to the Henderson–Hasselbalch equation^62^.

To determine the affinity of G-Flamp1, 1 μM of purified protein in HEPES buffer was mixed with varying concentrations of cAMP (0.001, 0.01, 0.1, 0.5, 1, 2, 5, 10, 25, 100, and 500 μM) or cGMP (0.01, 0.1, 0.5, 1, 2, 5, 10, 25, 100, 500, 1000, and 2000 μM). The fluorescence excited at 450 nm were recorded with an Infinite M1000 PRO fluorometer. The fluorescence change Δ*F*/*F*_0_ was plotted against the cAMP or cGMP concentrations and fitted by a sigmoidal binding function to determine the *K*_d_ and Hill coefficient^56^.

The association rate constant (*k*_on_) and dissociation rate constant (*k*_off_) between G-Flamp1 and cAMP were determined using Pro-data Chirascan 4.5.1840.0 software of the Chirascan spectrometer equipped with an SX20 Stopped-Flow accessory (Applied Photophysics Ltd). Briefly, 1.6 μM of protein solution was mixed 1:1 with cAMP of different concentrations (0.5, 1, 2, 5, 10, and 50 μM) and the fluorescence excited at 480 nm were measured with a 520/30 nm filter. The data were fitted using the following single-exponential function^63,64^: *F*(t) = *F*_0_ + *A*_obs_ × exp(−*k*_obs_ × *t*), where *F*(*t*) is the value of fluorescence increase at time *t*, *F*_0_ is the final value of fluorescence increase, *A*_obs_ is the amplitude of the exponentially decreasing part and *k*_obs_ is the observed first-order rate constant. The *k*_on_ and *k*_off_ were fitted using the following equation: *k*_obs_ = *k*_on_ × [cAMP] + *k*_off_, where [cAMP] is the concentrations of cAMP used. The association and dissociation half-time *t*_on_ and *t*_off_ were calculated as ln2/(*k*_on_ × [cAMP]) and ln2/*k*_off_, respectively.

To get the excitation wavelength-dependent brightness and Δ*F*/*F*_0_ under two-photon excitation, purified proteins were excited with wavelengths from 700 to 1000 nm with a 20 nm step size on a Nikon-TI two-photon microscope equipped with a Ti:sapphire laser and a 25 × 1.4 NA water immersion objective. Images were captured using NIS-Elements AR 4.30.01 software. The 495–532 nm fluorescence were collected and the intensities were then normalized to laser powers at different wavelengths.

### Crystallization and structure determination of G-Flamp1

The coding sequence of G-Flamp1 was cloned into pSUMO expression vector with 6× His and SUMO tags at the N-terminus. *E.coli* BL21 (DE3) pLysS cells were transformed with pSUMO-G-Flamp1 and grew on LB agar overnight at 34 °C. Colonies were expanded in LB media at 34 °C and induced at OD 0.6 with 0.1 mM IPTG for additional 3 h at 34 °C. The harvested cells were lysed with a high-pressure homogenizer at 1000 bar in binding buffer (20 mM Imidazole, 500 mM NaCl, 20 mM Tris-HCl, pH 7.5). The protein was purified on a Ni Sepharose 6 Fast Flow column (GE Healthcare) under gravity and eluted with the elution buffer (300 mM Imidazole, 500 mM NaCl, 20 mM Tris-HCl, pH 7.5). The elution was incubated with ULP1 protease and dialyzed against the dialysis buffer (100 mM NaCl, 10 mM β-ME, 20 mM Tris-HCl, pH 7.5) overnight at 4 °C and purified again on a Ni Sepharose 6 Fast Flow column to remove the 6×His and SUMO tags and ULP1 protease. After concentration, the flow-through was loaded on a Hiload 16/600 Superdex 200 pg column (GE Healthcare) in the dialysis buffer for further purification. Fractions containing purified protein were pooled, concentrated, and incubated with cAMP at 1:5 molar ratio for 1 h at 4 °C. Crystals were grown using the hanging drop vapor diffusion method with 2 μL protein solution (10 mg/mL) and 2 μL reservoir solution (40% v/v PEG 400, 100 mM Imidazole, pH 8.0). The mixture was equilibrated against 300 μL reservoir solution at 20 °C for 5 days. Crystals were flash-frozen for X-ray diffraction data collection. A data set was collected to 2.2 Å resolution at wavelength 1.0000 Å on beamline BL17B1 of the Shanghai Synchrotron Radiation Facility (SSRF). Data sets were processed with HKL3000 v716.1^65^. The structure was solved by molecular replacement method using Phaser 2.7.16 software^66^ implanted in the Phenix program suite 1.17.1^67^, with cpGFP (PDB: 3EVP) and mlCNBD (PDB: 3CLP) as search models. The model building was performed manually using the Coot 0.9^68^.

### Metadynamics molecular dynamics simulations

The crystal structure of cAMP-bound G-Flamp1 (PDB ID: 6M63) was used to perform the metadynamics MD simulations for the energy stable state(s) of both cAMP-bound and cAMP-free forms. The Schrödinger Protein Preparation Wizard (Maestro Version 13.0.135, MMshare version 5.6.135) was used for protein preparation^69^. For cAMP-bound form modeling, the waters beyond 3.0 Å were removed and the chromophore was set to be deprotonated. For the cAMP-free form modeling, the cAMP was further removed. Both cAMP-bound and cAMP-free systems were built using Desmond System builder (Schrödinger Release 2021-4, https://www.schrodinger.com/). The TIP3P solvent model was used, and the size of orthorhombic water box was 10 × 10 × 10 Å. Nine sodium ions and 0.15 M sodium chloride were added to neutralize the system. The OPLS4 force field was applied^70^ and the previously reported metadynamics procedure was used^71^. The system was relaxed for 1 ps before simulation. Simulation conditions in the isothermal-isobaric (NPT) ensemble were as follows: 1 bar of pressure, 300 K of temperature, 0.03 kcal/mol of initial Gaussian hill height, 0.09 ps interval, 0.05 Å Gaussian width, and 1.2 kcal/mol of well-tempered parameter. The Nose-Hoover chain was used for thermostat. PyMOL 2.4.2 was used for structural analysis. The two collective variables (CVs) shown in Supplementary Fig. 10 are two dihedral angles from the side chain of Trp 75.

### Cell culture, DNA transfection, and virus infection

HEK293T, HeLa, and CHO cells were acquired from ATCC and were maintained in DMEM (HEK293T and HeLa cells) or DMEM/F12 (CHO cells) supplemented with FBS (10% v/v) and penicillin/streptomycin (both at 100 units/mL) in a humidified incubator at 37 °C with 5% CO_2_. Plasmid transfections of cultured cells were performed according to the Lipofectamine 2000 protocol. Primary cortical neurons were prepared from embryonic day 16 (E16) BALB/c mice as previously described^72^ and kept in Neurobasal medium with B27 (2%) and penicillin/streptomycin (both at 100 units/mL). DIV (days in vitro) 7–9 neurons were infected with AAV8-CAG-G-Flamp1 virus prepared using PEG8000/NaCl solution and imaged at DIV13-18.

### Stable cell line generation and proliferation rate measurement

The CAG promoter and G-Flamp1 were inserted between two terminal inverted repeats for *piggyBac* transposase (PBase) in pPB-LR5 vector^73^ to make pPB-LR5-CAG-G-Flamp1. HEK293T cells in a 24-well plate were co-transfected with 1 μg of pCMV-hyperactive PBase^73^ and 1 μg of pPB-LR5-CAG-G-Flamp1, expanded for 1 week and then sorted for medium-brightness ones with a BD FACSAria III Cell Sorter (BD, USA). The proliferation rates of HEK293T control cells or cells expressing G-Flamp1 were measured using the Enhanced Cell Counting Kit-8 (Cat. No. C0041, Beyotime Biotechnology, Shanghai, China).

### Western blotting

Total protein of cells was extracted by radioimmunoprecipitation assay (RIPA) buffer (Beyotime Biotechnology, Shanghai, China) and protein concentrations were measured using BCA Protein Assay kit (Pierce, USA). Equal amounts of protein were separated by 4–10% SDS-PAGE, transferred on PVDF membranes, and immuno-detected with primary antibodies against pCREB and CREB. Signal detection was carried out on a ChemiDoc MP imaging system (Bio-Rad, Image Lab 6.0.1 Build 34 software) using the ECL kit (Cat. No. #32106, Pierce, USA).

### Wide-field fluorescence imaging of cAMP indicators in living cells

Wide-field imaging was performed on an Olympus IX83 microscope equipped with a 63 × 1.4 numerical aperture (NA) objective (HEK293T, HeLa and CHO cells) or a 20 × 0.75 NA objective (cultured neurons). The microscope was controlled using Micro-manager 1.4.21 software (https://micro-manager.org). Briefly, mammalian cells grown on glass-bottom dishes (Cat. No. #FD35-100, World Precision Instruments) were transfected with indicated plasmids and 24 h later serum-starved for 2–4 h. The culture medium was replaced with live cell imaging solution right before fluorescence imaging. Time-lapse images were captured every 15 s. The excitation and emission filters used for different sensors were as follows: ex 480/30 nm and em 530/30 nm for green sensors (GCaMP6s, cAMPr, Flamindo2 and G-Flamp1), ex 568/20 nm and em 630/50 nm for red sensors (jRCaMP1b, Pink Flamindo and R-FlincA), ex 441/20 nm and em 530/30 nm for G-Flamp1. The acquired images were analyzed using ImageJ 1.52p (NIH). Background-subtracted fluorescence was used to calculate fluorescence change Δ*F*/*F*_0_ that is defined as (*F* − *F*_0_)/*F*_0_, where *F*_0_ is the baseline signal before stimulation.

### Two-photon fluorescence imaging of cAMP indicators in living cells

Two-photon imaging was performed on a Nikon-TI two-photon microscope equipped with a Ti:sapphire laser and a 25 × 1.4 NA water immersion objective. In brief, mammalian cells grown on glass-bottom dishes were transfected with indicated plasmids and 24 h later serum-starved for 2–4 h. The culture medium was replaced with live cell imaging solution right before fluorescence imaging. Cells were excited with a 920 nm laser line and detected via a 495–532 nm filter. Time-lapse images were taken every 5 s and analyzed using ImageJ 1.52p (NIH). Background-subtracted fluorescence intensity was used to calculate Δ*F*/*F*_0_. The SNR was defined as the ratio of peak Δ*F*/*F*_0_ to the standard deviation of the basal Δ*F*/*F*_0_ fluctuation before stimulation.

### Fluorescence lifetime measurement of G-Flamp1 indicator in HEK293T cells

Fluorescence lifetime imaging microscopy (FLIM) was performed on the Leica TSC SP8 two-photon microscope (Leica) equipped with a Chameleon laser (Coherent, Inc.) and a 25 × 0.95 NA water immersion objective. The images were captured using Leica Application Suite X 3.5.6.21594 software. HEK293T cells expressing G-Flamp1 or G-Flamp1-mut were grown on glass-bottom dishes and the culture medium was replaced with live cell imaging solution right before fluorescence imaging. Cells were excited using a 920 nm laser line and fluorescence was collected with a 495–550 nm filter. The lifetime of cells was analyzed using the LAS X FLIM/FCS software (Leica Microsystems CMS GmbH).

### Brightness comparison of cAMP indicators in HEK293T cells

Fluorescent intensity of indicators was measured using an Infinite M1000 fluorometer or optical microscope. For fluorometer, HEK293T cells grown in 12-well plates were transfected with pCAG-G-Flamp1, pCAG-cMAPr, pCAG-Flamindo2, pCAG-Pink Flamindo, pCAG-R-FlincA, pCAG-GCaMP6s, pCAG-jRCaMP1b, pCAG-mEGFP or pCAG-mCherry construct separately using Lipofectamine 2000. 48 h later, the cells were washed once with PBS, suspended in live cell imaging solution (Cat. No. A14291DJ, Invitrogen) and transferred to a clear flat-bottom 96-well plate. The fluorescence was recorded under 488 nm excitation. For wide-field or two-photon microscopy, HEK293T cells on glass-bottom dishes were transfected with indicated constructs using Lipofectamine 2000. 48 h later, the culture medium was replaced with live cell imaging solution and fluorescence images were taken under 480/30 nm (one-photon) or 920 nm (two-photon) excitation.

### Two-photon imaging in zebrafish

cDNAs of G-Flamp1 (or G-Flamp1-mut) and NLS-mCherry (nuclear-localized mCherry) were subcloned into pTol2-UAS vector to make pTol2-UAS:G-Flamp1 (or G-Flamp1-mut)-T2A-NLS-mCherry, where T2A is a self-cleaving peptide. Plasmids above with Tol2 mRNA were co-injected into EF1α:Gal4 embryos at one-cell stage. At 52 h post-fertilization, the brain ventricle of larval zebrafish was injected with PBS or 120 μM Fsk and imaged with a BX61WI two-photon microscope (Olympus) equipped with a 25 × 1.05 NA water immersion objective. The excitation wavelength was 960 nm and 495–540 nm fluorescence was collected using FV10-ASW 4.2 software. The fluorescence intensities of cells pre- and post-treatment were extracted using ImageJ. Fluorescence change was calculated as Δ*F*/*F*_0_, where *F*_0_ was the average intensity before treatment.

### Two-photon imaging of transgenic flies

The coding sequence of G-Flamp1 was cloned into pJFRC28 (Addgene plasmid #36431). The vector was injected into embryos and integrated into attP40 via phiC31 by the Core Facility of Drosophila Resource and Technology (Shanghai Institute of Biochemistry and Cell Biology, Chinese Academy of Sciences). Stock 30Y-Gal4 (III) is a gift from Yi Rao lab (Peking University). Stock UAS-GFP (III) is a gift from Donggen Luo lab (Peking University). Flies UAS-G-Flamp1/+; 30Y-Gal4/+ and UAS-GFP/30Y-Gal4 were used. Flies were raised on standard cornmeal-yeast medium at 25 °C, with 70% relative humidity and a 12 h/12 h light/dark cycle.

Adult females within 2 weeks post-eclosion were used for in vivo imaging with a two-photon microscope FV1000 (Olympus) equipped with the Mai Tai Ti:Sapphire laser (Spectra-Physics) and a 25 × 1.05 NA water immersion objective (Olympus). The microscope was controlled using Fluoview 3.1a software. The excitation wavelength was 930 nm and a 495–540 nm emission filter was used. The sample preparation was similar as previously described^42^. Before and after odor stimulation, 1000 mL/min constant pure air was applied to the fly. During 1 s odor stimulation, 200 mL/min air containing isoamyl acetate (Cat. No. 306967, Sigma-Aldrich) mixed with 800 mL/min pure air was delivered to the fly. For electrical shock, 80 V 500 ms electrical stimulus was applied to the fly via copper wires attached to the abdomen. For Fsk application, the blood-brain barrier was carefully removed and Fsk was applied with a 100 μM final concentration. Customized Arduino code was used to synchronize the imaging and stimulation protocols. The sampling rate during odor stimulation, electrical shock stimulation and Fsk perfusion was 6.7, 6.7, and 1 Hz, respectively.

### Animals

All procedures for animal surgery and experimentation were conducted using protocols approved by the Institutional Animal Care and Use Committees at Shenzhen Institute of Advanced Technology-CAS, Peking University, and Institute of Neuroscience-CAS.

### Two-photon imaging in mice

AAV9-hSyn-G-Flamp1, AAV9-hSyn-G-Flamp1-mut and AAV9-hSyn-NES-jRGECO1a viruses were packaged at Vigene Biosciences (Jinan, China). Wild-type female C57 BL/6 J mice (6–8 weeks old) were anesthetized with an injection of Avertin or isoflurane (3% induction; 1–1.5% maintenance). The skin and skull above the motor cortex were retracted from the head and a metal recording chamber was affixed. ∼300 nL of AAV was injected into the motor cortex (AP, 1.0 mm relative to bregma; ML, 1.5 mm relative to bregma; depth, 0.5 mm from the dura). A 2 mm × 2 mm or 4 mm × 4 mm square coverslip was used to replace the skull. Three weeks after virus injection, wake mice were habituated for about 15 min in the treadmill-adapted imaging apparatus to minimize the potential stress effects of head restraining. The motor cortex at a depth of 100–200 μm below the pial surface was imaged using Prairie View 5.5.64.100 software of a Bruker Ultima Investigator two-photon microscope equipped with the Spectra-Physics Insight X3 and a 16 × 0.8 NA water immersion objective. 920 nm laser line was used for excitation of both green and red indicators. 490–560 nm and 570–620 nm filters were used for green and red fluorescence collection, respectively. The sampling rate was 1.5 Hz. For imaging analysis, we first corrected motion artifact using motion correction algorism (EZcalcium)^74^ and bleed-through between green and red channels using the spectral unmixing algorithm (see details in https://imagej.nih.gov/ij/plugins/docs/SpectralUnmixing.pdf). The fluorescence intensities of ROIs covering the somata were extracted using ImageJ software. Background-subtracted fluorescence intensity was used to calculate Δ*F*/*F*_0_. Correlations of the time series between cAMP and calcium signals were performed using Pearson correlation analysis with Matlab R2020a (MathWorks)^75^.

### Fiber photometry recording of cAMP signals in behaving mice

The AAV9-hSyn-G-Flamp1 virus was packaged at Vigene Biosciences (Jinan, China). Virus was unilaterally injected into NAc of adult C57BL/6N mice (male, >8 weeks old). During the surgery, mice were deeply anesthetized with isoflurane (RWD Life Science) and mounted on a stereotaxic apparatus (RWD Life Science). Approximately 300 nL of AAV2/9-hSyn-G-Flamp1 (titer 7.29 × 10^13^, 1:7 diluted with 1× PBS before use) was injected into the NAc (AP, +1.0 mm; ML, +1.5 mm; −3.9 mm from cortical surface) at a speed of 23 nL/injection (inter-injection interval 15–30 s) using a microinjection pipette injector (Nanoject II, Drummond Scientific). A 200 µm optic fiber (Thorlabs, FT200UMT) housed in a ceramic ferrule was implanted to the same coordinate two weeks later and a stainless steel headplate was affixed to the skull using machine screws and dental cement. After recovery (>5 days), the mouse was water-restricted to achieve 85–90% of normal body weight and prepared for behavior training. Mice were trained on an auditory conditioning task, in which three auditory cue - outcome pairs (or CS-US pairs; 8 kHz pure tone → 9 µL water; white noise → brief air puff on face; 2 kHz pure tone → nothing) were randomly delivered with 10–20 s randomized inter-trial intervals. The duration of each sound is 1 s and sound intensity was calibrated to 70 dB. The outcomes were delivered 1 s after offset of each sound. The behavioral setup consisted of a custom-built apparatus allowing head fixation of mice. Licking behavior was detected when the tongue of the mouse contacted the water delivery tube. Lick signal was processed in an Arduino UNO board with custom code and sent digitally to the training program (written in Matlab) via a serial port. Water delivery was precisely controlled by a stepping motor pump and air puff (15 psi, 25 ms) was controlled by a solenoid valve. The timing of the pump and valve was controlled by the same Arduino UNO board used for lick detection, which also provides synchronization between the training program and data accusation system (RZ2, TDT). During the first two days of each training, the outcomes were delivered without the prediction cues. To record the fluorescence signal from the cAMP sensor, an optic fiber (Thorlabs, FT200UMT) was attached to the implanted ferrule via a ceramic sleeve. The photometry rig was constructed using parts from Doric Lens, which includes a fluorescence optical mini cube (FMC4_AE(405)_E(460–490)_F(500–550)_S), a blue led (CLED_465), a led driver (LED_2) and a photo receiver (NPM_2151_FOA_FC). During recording, a software lock-in detection algorithm (modulation frequency: 459 Hz; low-pass filter for demodulated signal: 20 Hz, 6th order) was implemented in a real-time processor (RZ2 with fiber photometry gizmo in Synapse software). The intensity of excitation light was measured as ∼70 µW from tip of the optical fiber. The photometry data was stored using a sampling frequency of 1017 Hz. To analyze the recording data, we first binned the raw data to 10.17 Hz (down-sampled by 100), and then fitted the binned data with a 2nd order exponential function using Matlab Curve Fitting Tool. The fitting data was then subtracted from the binned data in order to remove the baseline drift resulting from photo-bleaching, and baseline corrected data was converted to z-score for further analysis. To analyze CS- or US-evoked changes in cAMP signals, we aligned each trial to the auditory cue onset and calculated the peri-stimulus time histogram (PSTH). To compare PSTH changes during different phases of the training, we used data from the 2nd day as naïve, the 5th day as trained and the 11th day as well-trained. Response to CS was defined as peak of the PSTH between CS onset to US onset and response to US was calculated accordingly using data from US onset to 2 s after US onset. To examine the contribution of dopamine signaling to the cAMP signals in NAc during spontaneous wakefulness, a potent dopamine receptor antagonist, SCH23390 (ab120597, Abcam; 0.2 mg/kg in 100 µL 0.9 % NaCl, i.p.) was administered to mice after tens of minutes of baseline were recorded. To be noted, recordings were not interrupted during the i.p. injection. Each mouse used for analysis had been administered with both SCH23390 and vehicle (100 µL 0.9% NaCl, i.p.), but only one of the solutions was used each single day. To quantify the change in cAMP signals, we take the mean of the z-score transformed signal to get Fig. 5j.

### Statistics

The statistical significances between groups were determined using two-tailed Student’s *t*-tests, One-way ANOVA tests (Fig. 3g, h) or Post hoc Tukey’s tests (Fig. 5g, h) with OriginPro 9.1 (OriginLab). *P < 0.05, **P < 0.01, ***P < 0.001 and NS (not significant) for *P* > 0.05.

### Reporting summary

Further information on research design is available in the Nature Research Reporting Summary linked to this article.

## Supplementary information

Supplementary Information

Reporting Summary

## Data Availability

The atomic coordinates and structure factors of the G-Flamp1 (no RSET peptide) and cAMP complex have been deposited in the Protein Data Bank with PDB ID code 6M63. Other PDB files used in this study are: 1VP6, 3WLD, 3EVP, 3CLP, 1RL3, 1U12, 2BYV, 4JV4, 3CF6, 6DGV, and 5UKG (https://www.rcsb.org/structure/1VP6, https://www.rcsb.org/structure/3WLD, https://www.rcsb.org/structure/3EVP, https://www.rcsb.org/structure/3CLP, https://www.rcsb.org/structure/1RL3, https://www.rcsb.org/structure/1U12, https://www.rcsb.org/structure/2BYV, https://www.rcsb.org/structure/4JV4, https://www.rcsb.org/structure/3CF6, https://www.rcsb.org/structure/6DGV and https://www.rcsb.org/structure/5UKG). Plasmids expressing G-Flamp1 and G-Flamp1-mut have been deposited to Addgene (https://www.addgene.org/188567, https://www.addgene.org/188568, https://www.addgene.org/188569, https://www.addgene.org/188570). Source data are provided with this paper.

## Source data

Source Data

## Code availability

The custom Arduino code for stimulation and two-photon imaging in *Drosophila*, the custom MATLAB and Arduino codes for fiber photometry in mice, and the custom MATLAB code for data analysis can be made available upon reasonable request.
