# Supplementary material for: A high-performance genetically encoded fluorescent indicator for in vivo cAMP imaging
Source: Nat Commun. 2022 Sep 12;13:5363. doi: 10.1038/s41467-022-32994-7 (PMC9468011; doi:10.1038/s41467-022-32994-7)
Supplement: Supplementary file 1 — Supplementary Information [file 41467_2022_32994_MOESM1_ESM.pdf]

A high-performance genetically encoded fluorescent indicator for *in vivo* cAMP imaging

Liang Wang, Chunling Wu, Wanling Peng, Ziliang Zhou, Jianzhi Zeng, Xuelin Li, Yini Yang, Shuguang Yu, Ye Zou, Mian Huang, Chang Liu, Yefei Chen, Yi Li, Panpan Ti, Wenfeng Liu, Yufeng Gao, Wei Zheng, Haining Zhong, Shangbang Gao, Zhonghua Lu, Pei-Gen Ren, Ho Leung Ng, Jie He, Shoudeng Chen, Min Xu, Yulong Li, Jun Chu

Contents:

1. Supplementary Figures

2. Supplementary Tables

3. Supplementary References

1. Supplementary Figures

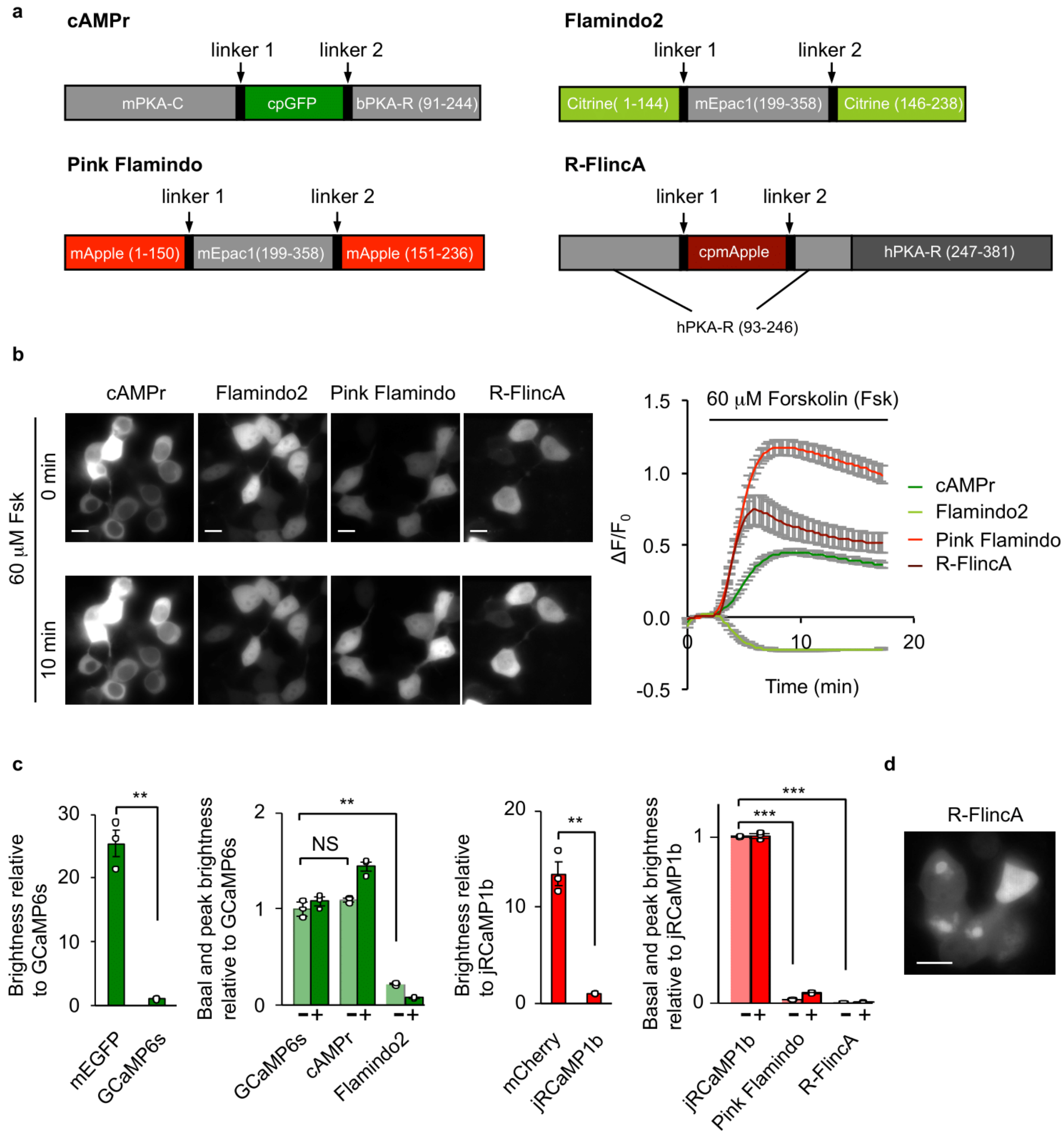

Supplementary Fig. 1 Key properties of previously reported single FP-based cAMP sensors in HEK293T cells at 37°C.

(a) Schematic of cAMPr, Flamindo2, Pink Flamindo and R-FlincA sensors. PKA-C and PKA-R represent PKA catalytic and regulatory subunit, respectively. mPKA, bPKA and hPKA are mouse, bovine and human PKA, respectively. mEpac1 is mouse Epac1. GFP,

Citrine and mApple are fluorescent proteins. In R-FlincA, cpmApple was inserted into the first CNBD of PKA-R.

(b) Representative fluorescence images (left) and traces of $\Delta F/F_0$ (right) of cAMP sensors in response to 60 μM Forskolin (Fsk) in HEK293T cells. Notably, the image contrasts for different sensors were different to render fluorescence visible. Data are shown as mean ± SEM. n = 33 cells (cAMPr), 42 cells (Flamindo2), 34 cells (Pink Flamindo) and 18 cells (R-FlincA) from 3 cultures for each sensor. Scale bars: 10 μm.

(c) Basal and peak brightness of the green cAMP sensors (cAMPr and Flamindo2) and red cAMP sensors (Pink Flamindo and R-FlincA) in HEK293T cells before and 15 min after 60 μM Fsk stimulation. Brightness of green and red cAMP sensors were normalized to those of the green calcium sensor GCaMP6s and the red calcium sensor jRCaMP1b, respectively. The brightness of GFP and mCherry were also normalized to GCaMP6s and jRCaMP1b, respectively. Data are shown as mean ± SEM. n = 3 wells from 12-well plates for each sensor. Two-tailed Student’s *t*-tests were performed. $P = 0.008$ between mEGFP and GCaMP6s, $P = 0.2039$ between GCaMP6s and cAMPr, $P = 0.0035$ between GCaMP6s and Flamindo2. $P = 0.0097$ between mCherry and jRCaMP1b, $P = 8.4 \times 10^{-8}$ between jRCaMP1b and Pink Flamindo2, $P = 8.7 \times 10^{-6}$ between jRCaMP1b and R-FlincA. The minus and plus signs denote without and with Fsk treatment, respectively.

(d) R-FlincA formed puncta in HEK293T cells after 48 h transfection. Representative image from 3 independent experiments is shown. Scale bar: 10 μm. $^{***}P < 0.001$, $^{**}P < 0.01$ and NS, not significant.

Source data are provided as a Source Data file.

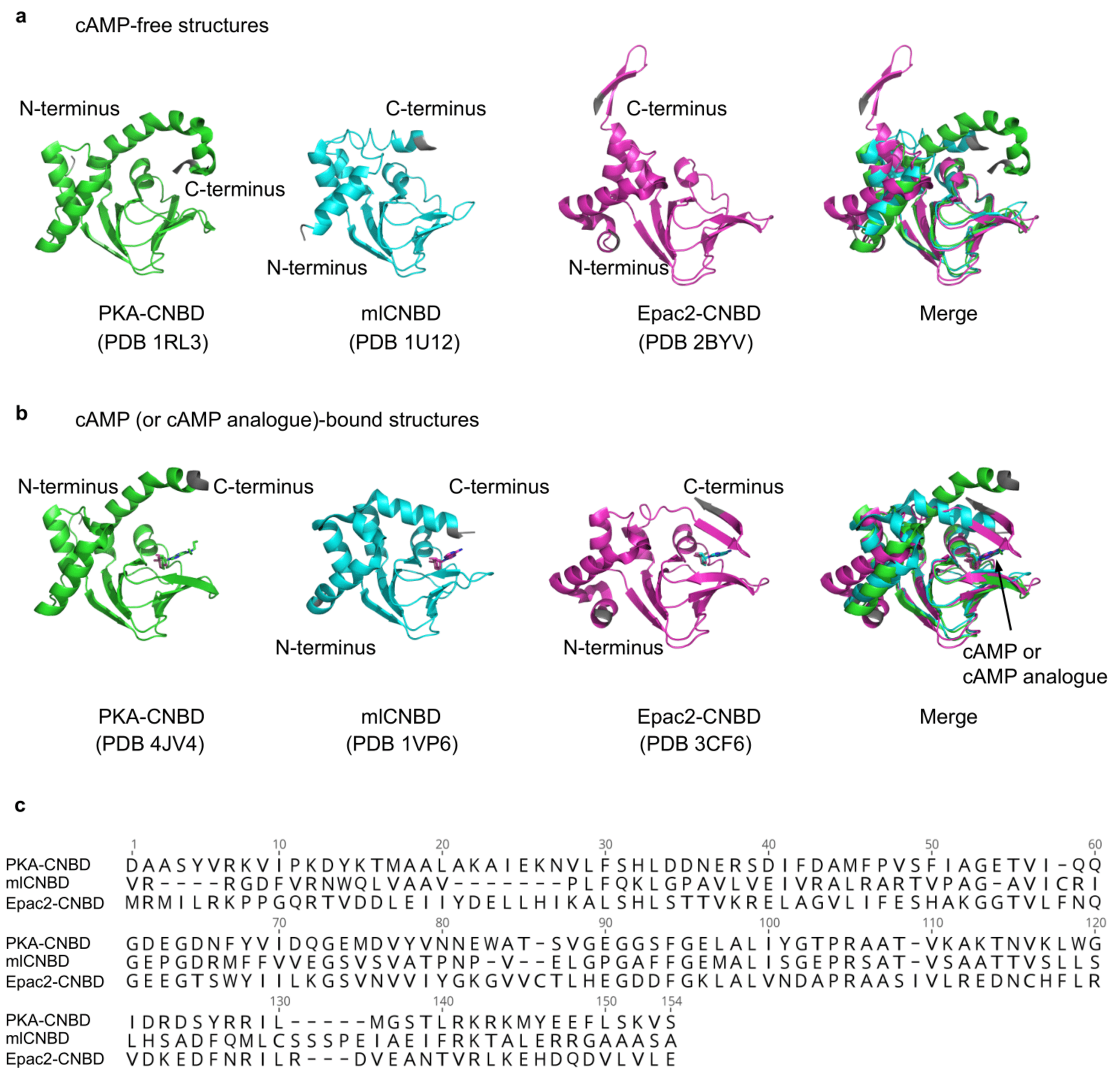

Supplementary Fig. 2 Structure and amino acid sequence alignments of CNBDs from bovine PKA, mouse Epac2 and bacterial MlotiK1 channel.

(a) Structures of different cAMP-free CNBDs.

(b) Structures of different cAMP (or its analogue)-bound CNBDs. cAMP or its analogue molecules are shown as stick models. Protein termini are highlighted in grey.

(c) Protein sequence alignment of CNBDs.

a

Evolution procedure	Sensor property (sensor variant)	Mutation
Step 1<br>Insertion site optimization	Responsive sensor<br>(G-Flamp0.1)	None
Step 2<br>cpGFP optimization<br>(site-directed mutagenesis)	Brightness enhancement<br>(G-Flamp0.2)	G-Flamp0.1 + S30R/Y39N/<br>S72A/F99V/N105T/K153T/I171V in GFP
Step 3<br>Linker optimization	Dynamic range improvement<br>(G-Flamp0.5)	G-Flamp0.2 + linker mutations<br>(linker 1 = WG, linker 2 = RV)
Step 4<br>cpGFP optimization<br>(random mutagenesis)	Brightness enhancement and<br>dynamic range improvement<br>(G-Flamp0.7)	G-Flamp0.5 + P285N in mlCNBD,<br>D173G in GFP
Step 5<br>Binding pocket optimization	Higher specificity<br>(G-Flamp1)	G-Flamp0.7 + S308V in mlCNBD

b

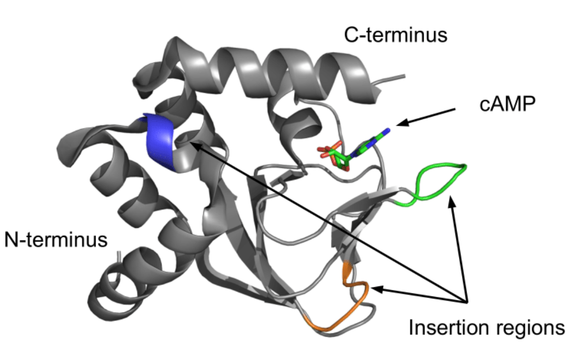

c

Insertion site	$\Delta F/F_0$ (excited at 488 nm)
Gln237-Lys238	0.012
Lys238-Leu239	-0.021
Ala283-Thr284	-0.004
Thr284-Pro285	-0.009
**Pro285-Asn286 (G-Flamp0.1)**	**-0.258**
Asn286-Pro287	0.076
Pro287-Val288	-0.126
Ala313-Ala314	0.009
Ala314-Thr315	-0.034
Thr315-Thr316	-0.005
Thr316-Val317	0.014

d

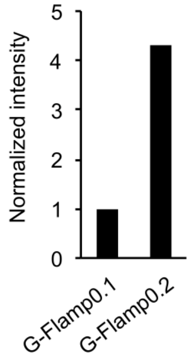

e

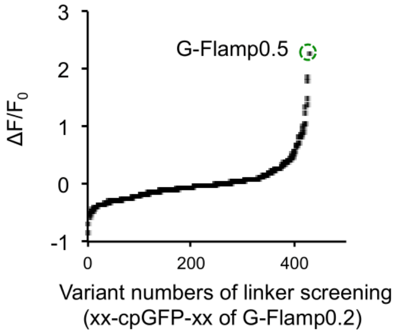

f

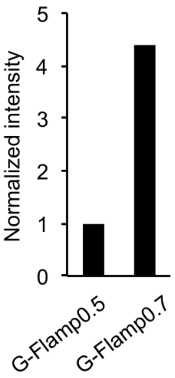

g

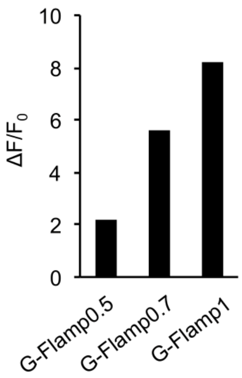

Supplementary Fig. 3 Evolution of G-Flamp1.

(a) Five-step directed evolution procedure of G-Flamp1.

(b) Three insertion regions tested are highlighted in blue, green and orange in mlCNBD's structure (PDB 1VP6).

(c) $\Delta F/F_0$ of 11 G-Flamp variants with different insertion sites in response to 500 μM cAMP. The variant with the insertion site between Pro285 and Asn286 (named G-Flamp0.1) showed the largest fluorescence change. The color coding matches the one in **b**.

(d) The brightness of G-Flamp0.1 and G-Flamp0.2 in bacterial cells cultured overnight at 34°C.

(e) $\Delta F/F_0$ of 427 G-Flamp0.2 variants with different linkers in response to 500 μM cAMP. The variant with the linkers 'WG' and 'RV' (named G-Flamp0.5) showed the greatest fluorescence change. Source data are provided as a Source Data file.

(f) The brightness of G-Flamp0.5 and G-Flamp0.7 in bacterial cells cultured overnight at 34°C.

(g) $\Delta F/F_0$ of G-Flamp0.5, G-Flamp0.7 and G-Flamp1 under excitation at 488 nm.

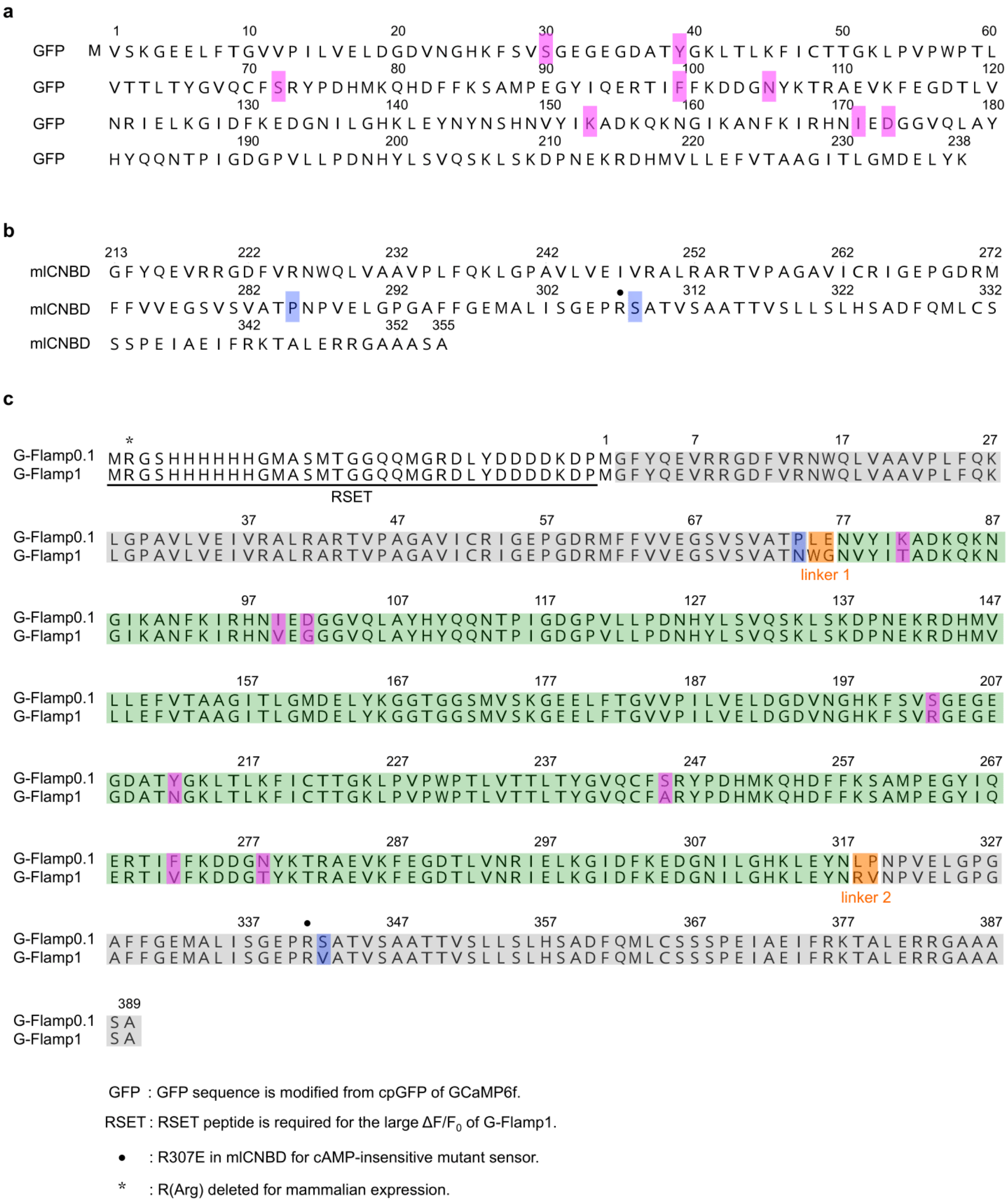

Supplementary Fig. 4 Protein sequences of GFP, mlCNBD, G-Flamp0.1 and G-Flamp1.

(a-b) Protein sequences of GFP and mlCNBD. The numberings of GFP and mlCNBD are according to PDB 2Y0G and 1VP6, respectively. Modified amino acid residues in G-Flamp1 sensor are highlighted in magenta and blue.

(c) Sequence alignment of full-length G-Flamp0.1 and G-Flamp1. The numbering is according to PDB 6M63. Modified amino acid residues are highlighted in magenta, blue and orange. Note the amino acid Arg immediately after the initiator methionine in G-Flamp1 was deleted for mammalian expression. Source data are provided as a Source Data file.

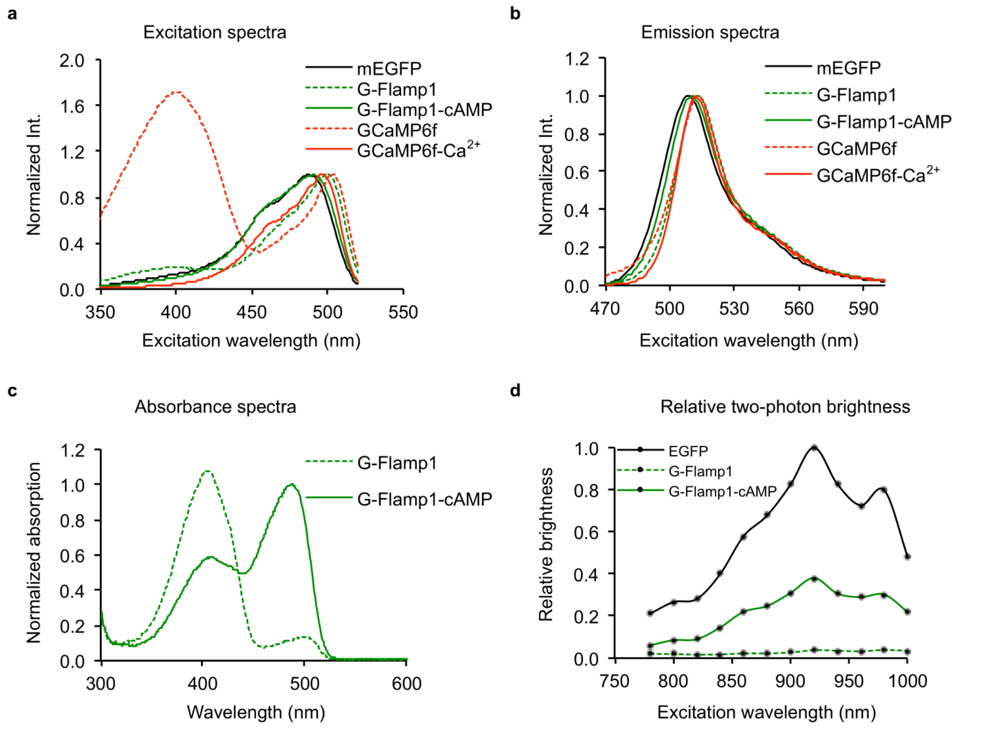

Supplementary Fig. 5 Fluorescence and absorption spectra of purified G-Flamp1, mEGFP and GCaMP6f.

(a-b) Excitation (a) and emission (b) spectra of mEGFP, cAMP-free G-Flamp1, cAMP-bound G-Flamp1, calcium-free GCaMP6f and calcium-bound GCaMP6f.

(c) Absorption spectra of 20 μM purified G-Flamp1 in HEPES buffer in the presence or absence of 500 μM cAMP.

(d) Relative brightness at different excitation wavelengths under two-photon excitation.

Source data are provided as a Source Data file.

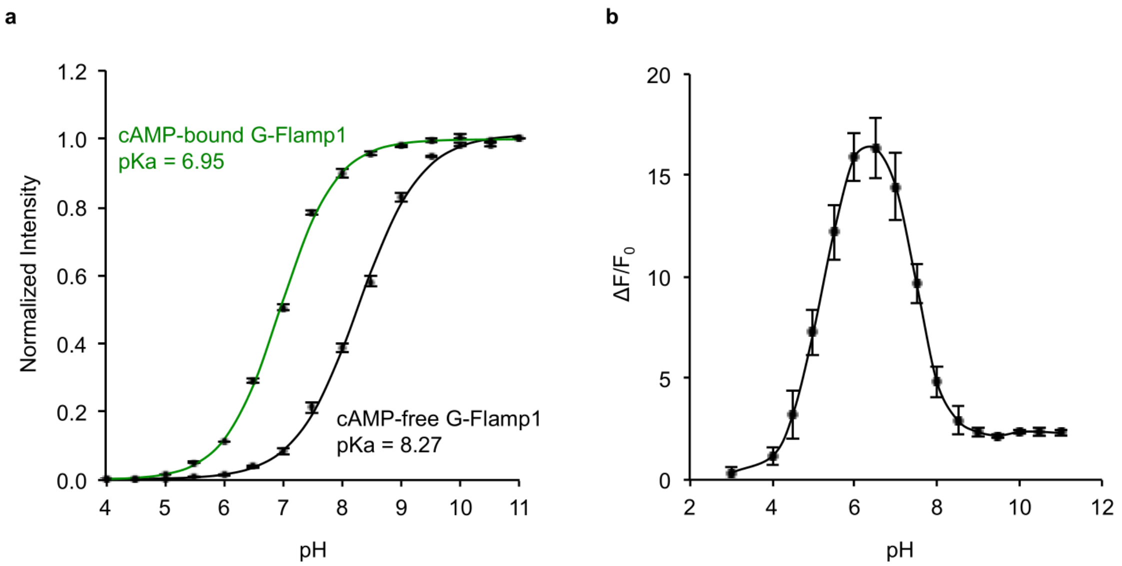

Supplementary Fig. 6 pH-dependent fluorescence and fluorescence change of purified G-Flamp1.

(a) Normalized fluorescence of purified G-Flamp1 (2 μM) at various pH values in the presence or absence of 500 μM cAMP. Fitted data are shown as solid lines.

(b) $\Delta F/F_0$ of purified G-Flamp1 (2 μM) in buffers with different pH values.

Data are presented as mean ± SEM. n = 3 independent experiments. Source data are provided as a Source Data file.

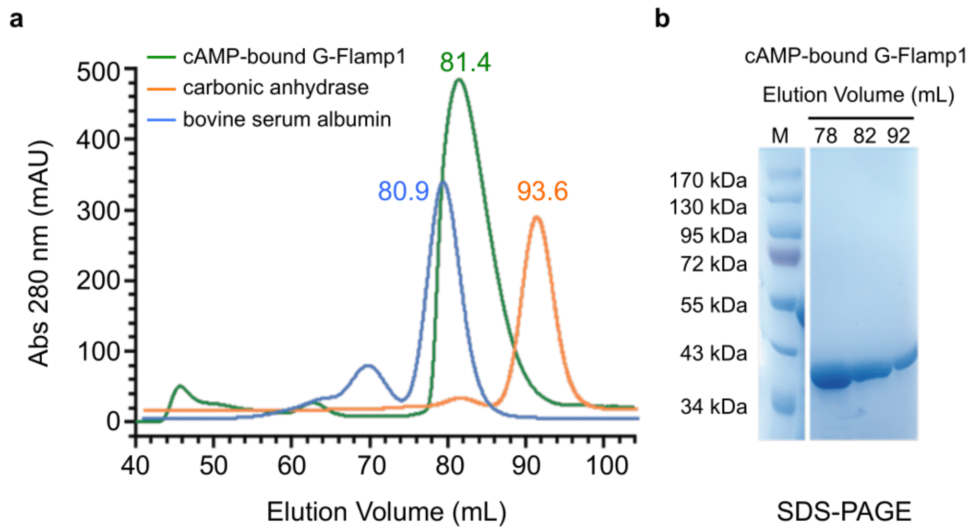

Supplementary Fig. 7 Gel filtration chromatography of G-Flamp1.

(a) The size-exclusion chromatogram for the cAMP-bound G-Flamp1 indicator without the RSET peptide (42.6 kDa) and standard molecular weight proteins (carbonic anhydrase, 29 kDa; bovine serum albumin, 66 kDa). 850 μL of 29.8 mg/mL (0.7 mM) G-Flamp1 was loaded into the column (Hiload 16/600 Superdex 200 pg column, GE Healthcare).

(b) SDS-PAGE of purified cAMP-bound G-Flamp1 using 10% gel. All eluted fractions showed specific bands with a molecular weight of ~43 kDa. Representative image from 3 independent experiments is shown.

Source data are provided as a Source Data file.

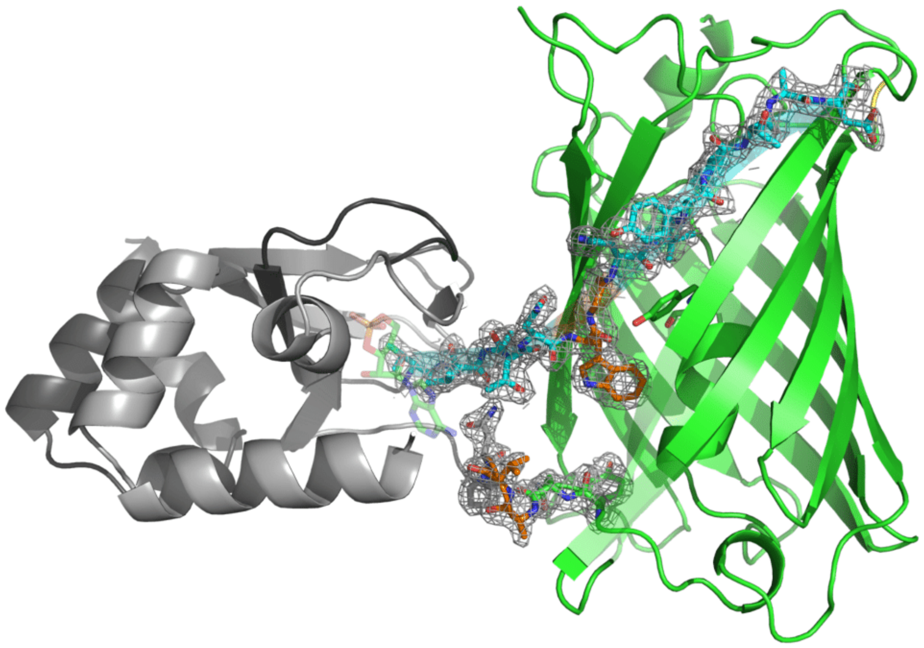

Supplementary Fig. 8 Crystal structure of cAMP-bound G-Flamp1 (PDB: 6M63) with electron density on both two linkers and their neighboring residues. The mesh depicts electron density in the 2Fo−Fc map contoured to 1.2 sigma within 2.0 Å of the atoms displayed in stick form.

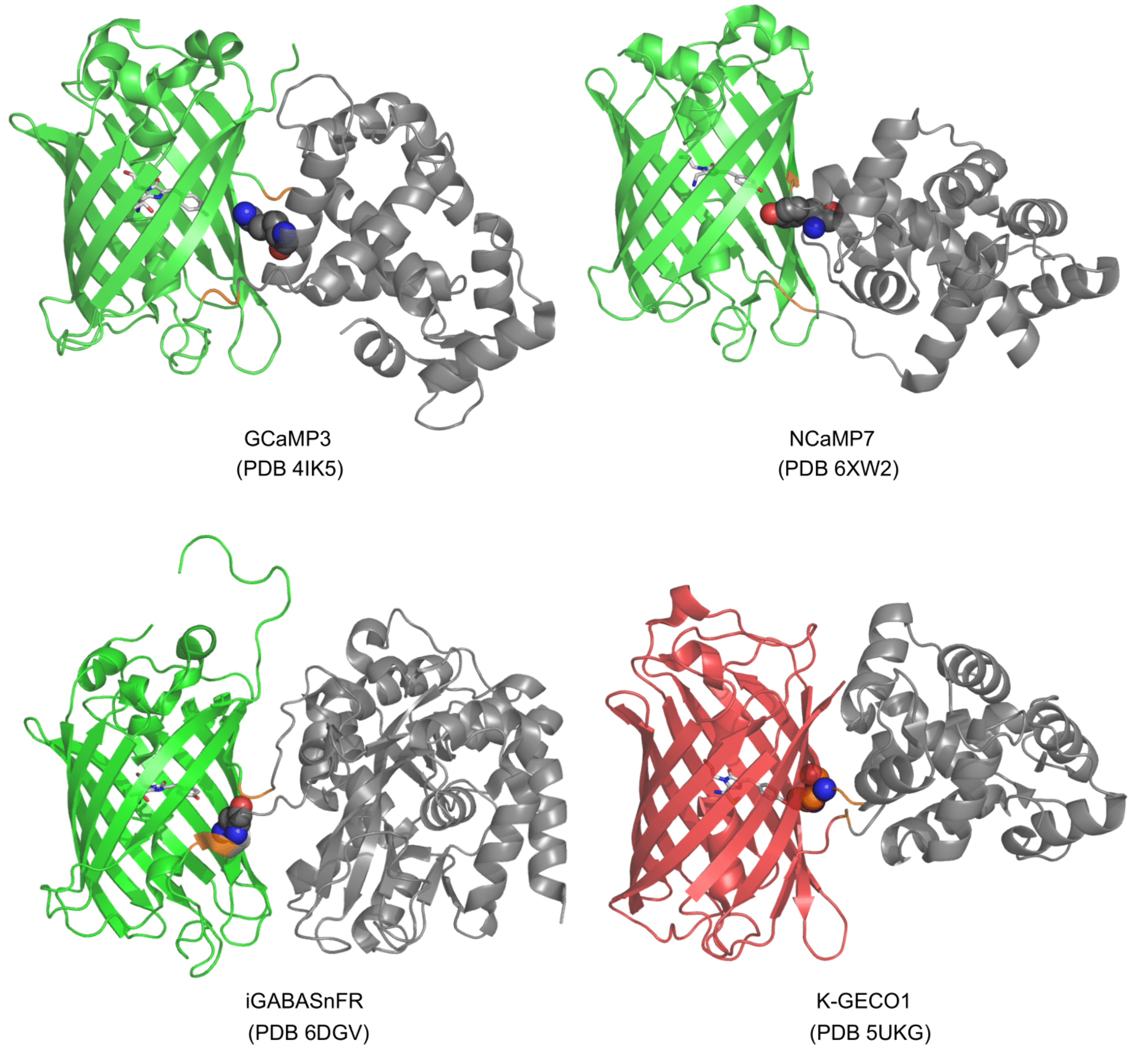

Supplementary Fig. 9 Linker conformation and the interactions between key residues and chromophore in other single-FP indicators. FPs, linkers and sensing domains are marked in green/red, orange and grey, respectively. All chromophores in the FP are shown as stick and amino acid residues interacting with the phenolic oxygen of the chromophore are shown as sphere. In iGABASnFR, the linker 2 folds as α-helix. Unlike GCaMP3 and NCaMP7, in which the fluorescence modulation is dependent on

the interactions with residues of CaM, the fluorescence change in K-GECO1 is mediated by a residue from linker 1.

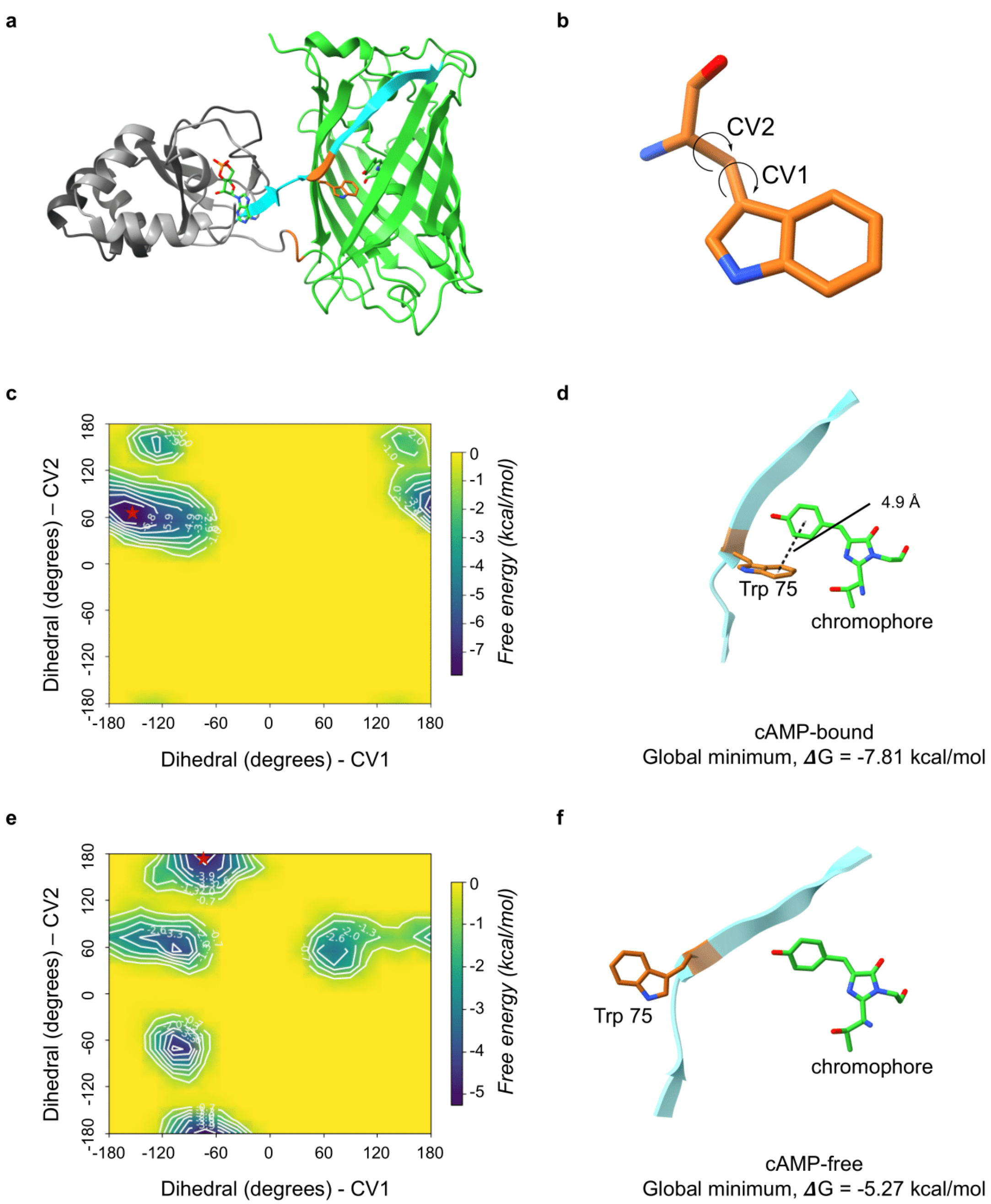

Supplementary Fig. 10 Stable cAMP-bound and cAMP-free structures predicted by the metadynamics molecular dynamics simulations.

(a) The simulated cAMP-bound G-Flamp1 structure at global minimum.

(b) The two dihedral angles picked for collective variables (CVs).

(c) Free energy landscape of the cAMP-bound form at 300 K. The red star shows the global minima (lowest-energy pose) and its free energy was -7.81 kcal/mol.

(d) The cAMP-bound conformation at global minimum. The side chain of Trp 75 is close to the chromophore. There is a pi-stacking interaction between the side chain of Trp 75 and the chromophore.

(e) Free energy landscape of cAMP-free form at 300K. The red star shows the global minima (lowest-energy pose) and its free energy was -5.27 kcal/mol.

(f) The cAMP-free conformation at global minimum. The side chain of Trp 75 moves away from the chromophore.

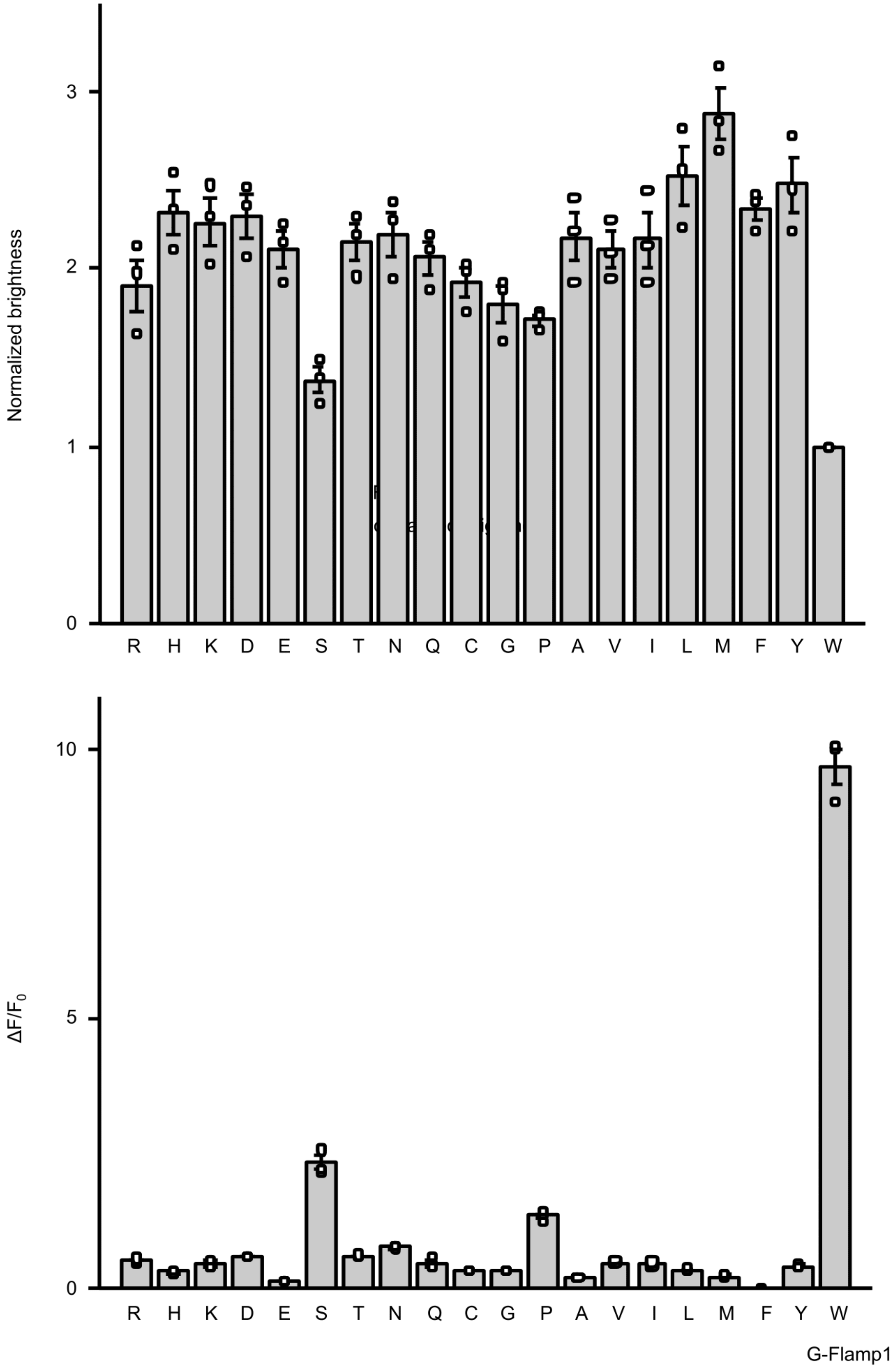
Normalized brightness
0
1
2
3
R H K D E S T N Q C G P A V I L M F Y W
ΔF/F0
0
5
10
R H K D E S T N Q C G P A V I L M F Y W
G-Flamp1

Supplementary Fig. 11 Saturation mutagenesis of Trp75 in G-Flamp1 sensor. Basal brightness (up) and $\Delta F/F_0$ (bottom) for each variant were shown. Error bars indicate SEM of the mean from 3 independent experiments. Source data are provided as a Source Data file.

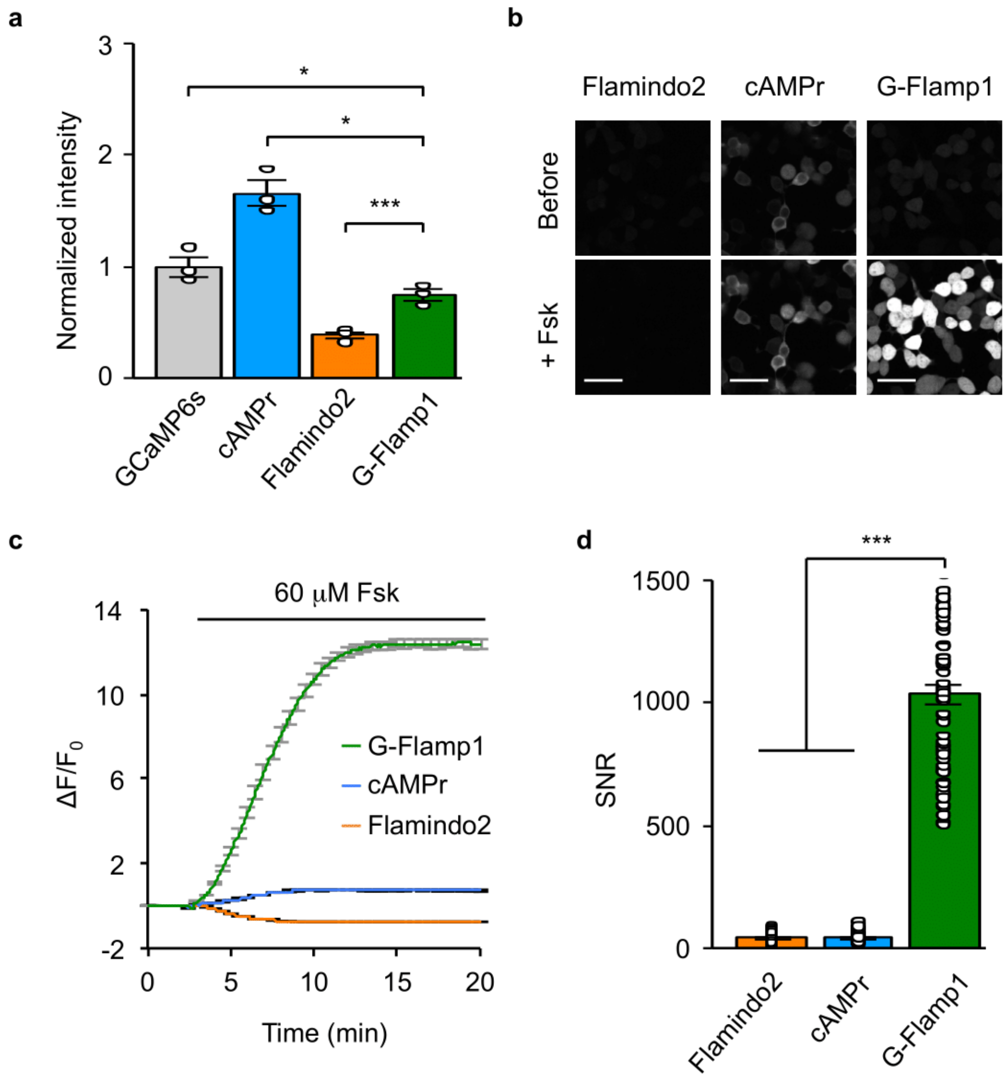

Supplementary Fig. 12 Performance of G-Flamp1 in HEK293T cells under two-photon imaging.

(a) Brightness comparison of three different green cAMP sensors (cAMPr, Flamindo2 and G-Flamp1) and GCaMP6s. Images were taken after 48 hours transfection under two-photon excitation (920 nm). n = 3 cultures for each sensor. Two-tailed Student's *t*-tests were performed. P = 0.044, 0.017 and 1.9×10^{-4} between G-Flamp1 and GCaMP6s, cAMPr and Flamindo2, respectively.

(b-c) Representative two-photon fluorescence images (b) and traces of $\Delta F/F_0$ (c) of HEK293T cells expressing cAMP sensors in response to 60 μM Fsk. n = 76 cells (Flamindo2), 35 cells (cAMPr) and 64 cells (G-Flamp1) from 2 separate experiments. Scale bars: 50 μm.

(d) Signal-to-noise ratio (SNR) of different sensors in (c). Two-tailed Student's *t*-tests were performed. $P = 6.1 \times 10^{-33}$ between G-Flamp1 and Flamindo2, and $P = 4.7 \times 10^{-33}$ between G-Flamp1 and cAMPr.

All data are shown as mean ± SEM in **a, c** and **d.** ***$P < 0.001$ and *$P < 0.05$. Source data are provided as a Source Data file.

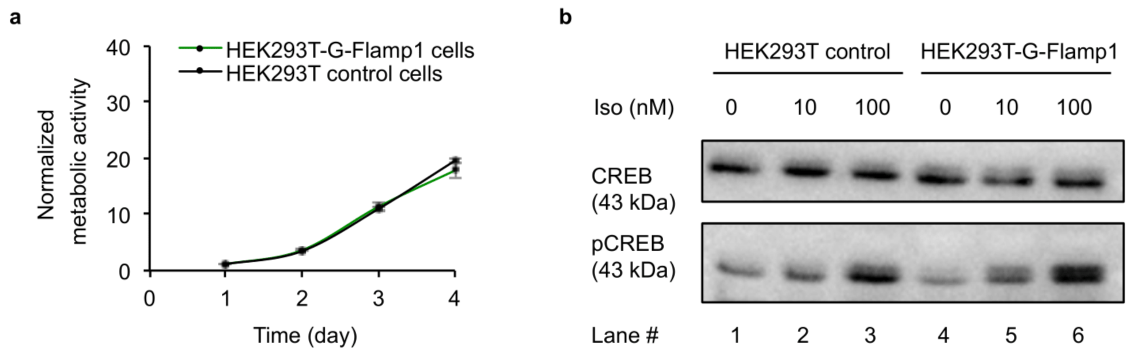

Supplementary Fig. 13 Effects of G-Flamp1 expression on HEK293T proliferation and cAMP signaling.

(a) Proliferation rates of HEK293 cells (control) and stable HEK293T cells expressing G-Flamp1 (HEK293T-G-Flamp1) were measured using the CCK-8 assay. Data are shown as mean ± SEM from 3 independent experiments.

(b) Western blot analysis of phosphorylated CREB (pCREB) in cells induced by 10 nM or 100 nM Iso for 1 hour. Representative images from 3 separate experiments are shown. Lanes 1-3 and 4-6 were the lysates of serum-starved control and stable HEK293T cells expressing G-Flamp1 (HEK293T-G-Flamp1), respectively.

Source data are provided as a Source Data file.

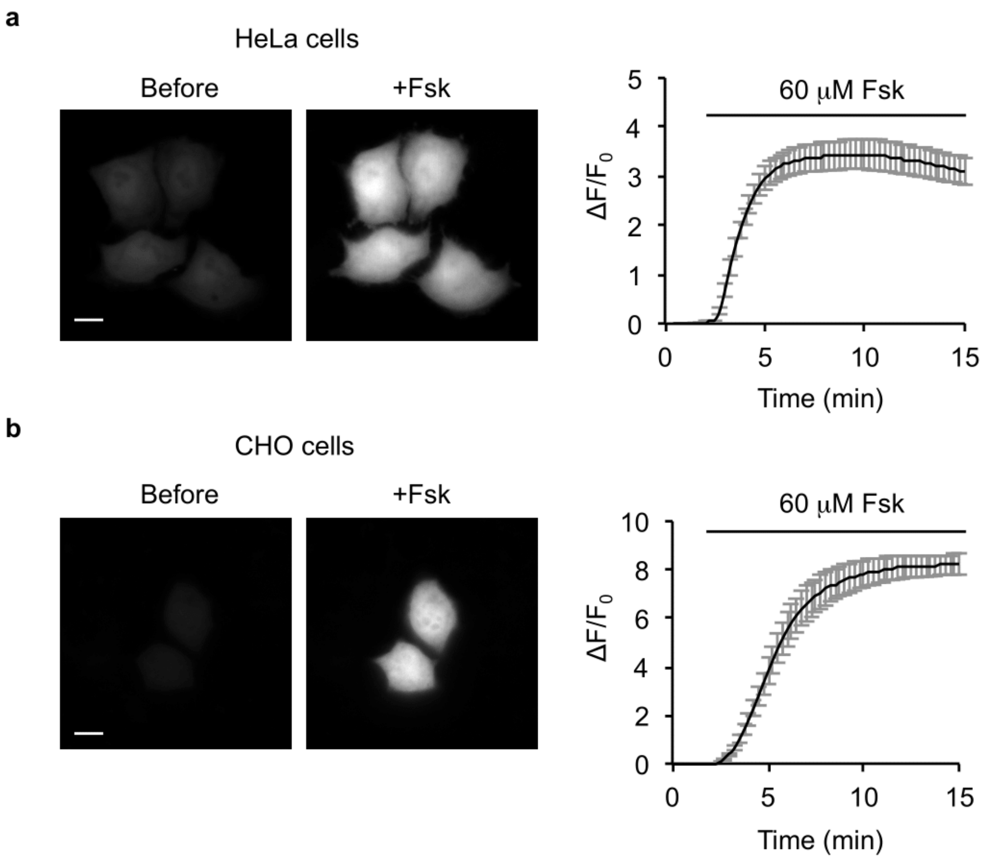

Supplementary Fig. 14 $\Delta F/F_0$ of G-Flamp1 in HeLa and CHO cells.

(a) Representative fluorescence images (left) and $\Delta F/F_0$ traces (right) of HeLa cells expressing G-Flamp1 in response to 60 μM Fsk. n = 18 cells from 2 cultures.

(b) Same as **a** except that the mammalian cell line used was CHO. n = 13 cells from 6 cultures.

Data are shown as mean ± SEM. Scale bars: 10 μm. Source data are provided as a Source Data file.

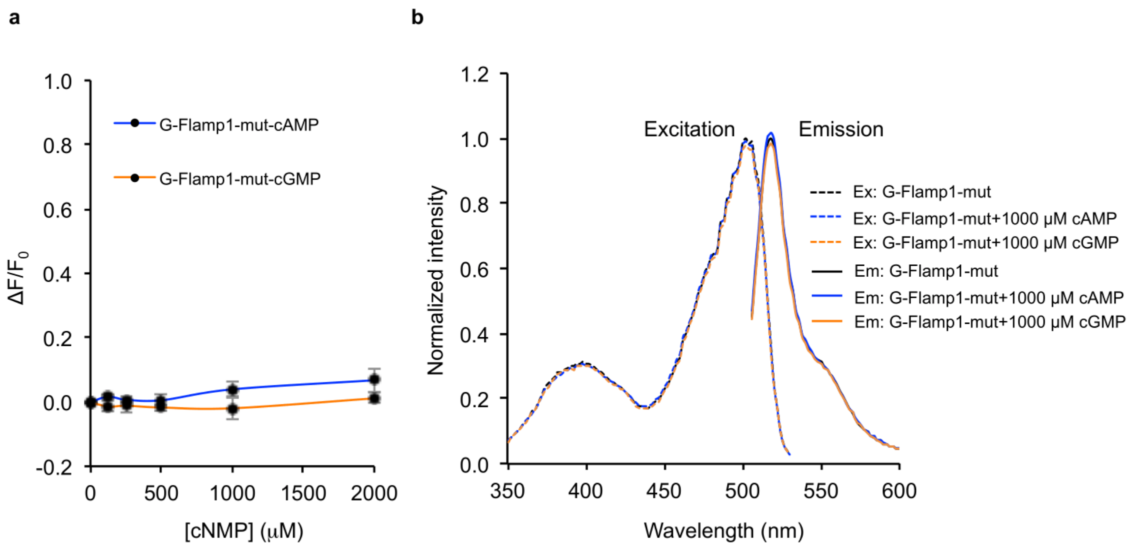

Supplementary Fig. 15 The responses of G-Flamp1-mut to cAMP or cGMP *in vitro*.

(a) $\Delta F/F_0$ of purified G-Flamp1-mut in response to various concentrations of cAMP or cGMP. The fluorescence under excitation at 450 nm was collected. Data are shown as mean ± SEM from 3 independent experiments.

(b) Excitation and emission spectra of purified G-Flamp1-mut without cAMP or cGMP (black), with 1000 μM cAMP (blue) and with 1000 μM cGMP (orange). Ex and Em stand for excitation and emission, respectively.

Source data are provided as a Source Data file.

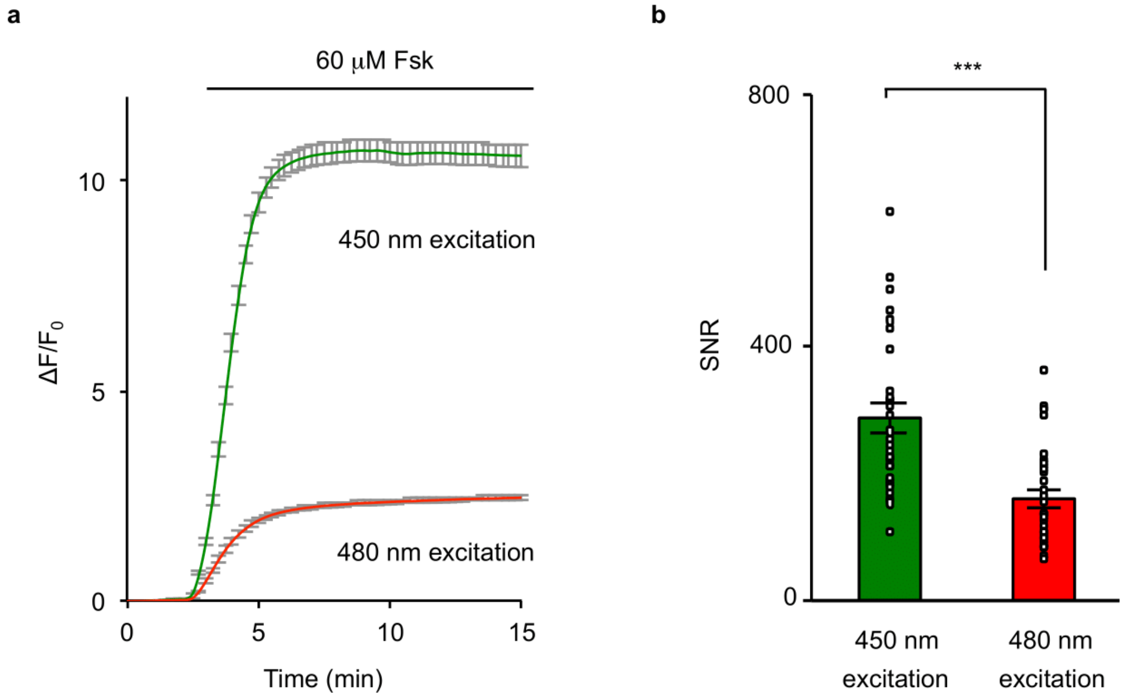

Supplementary Fig. 16 $\Delta F/F_0$ and signal-to-noise ratio (SNR) of G-Flamp1 in HEK293T cells under 450 nm and 480 nm excitations.

(a) Representative traces of $\Delta F/F_0$ of HEK293T cells stably expressing G-Flamp1 in response to 60 μM Fsk. n = 30 cells (450 nm excitation) and 32 cells (480 nm excitation) from 3 cultures.

(b) SNRs of G-Flamp1 under 450 nm and 480 nm excitations. n = 30 cells (450 nm excitation) and 32 cells (480 nm excitation) over 3 independent experiments. Two-tailed Student's *t*-test was performed. $P = 1.1 \times 10^{-5}$ between the two groups.

All data are shown as mean ± SEM in **a** and **b.** ***$P < 0.001$. Source data are provided as a Source Data file.

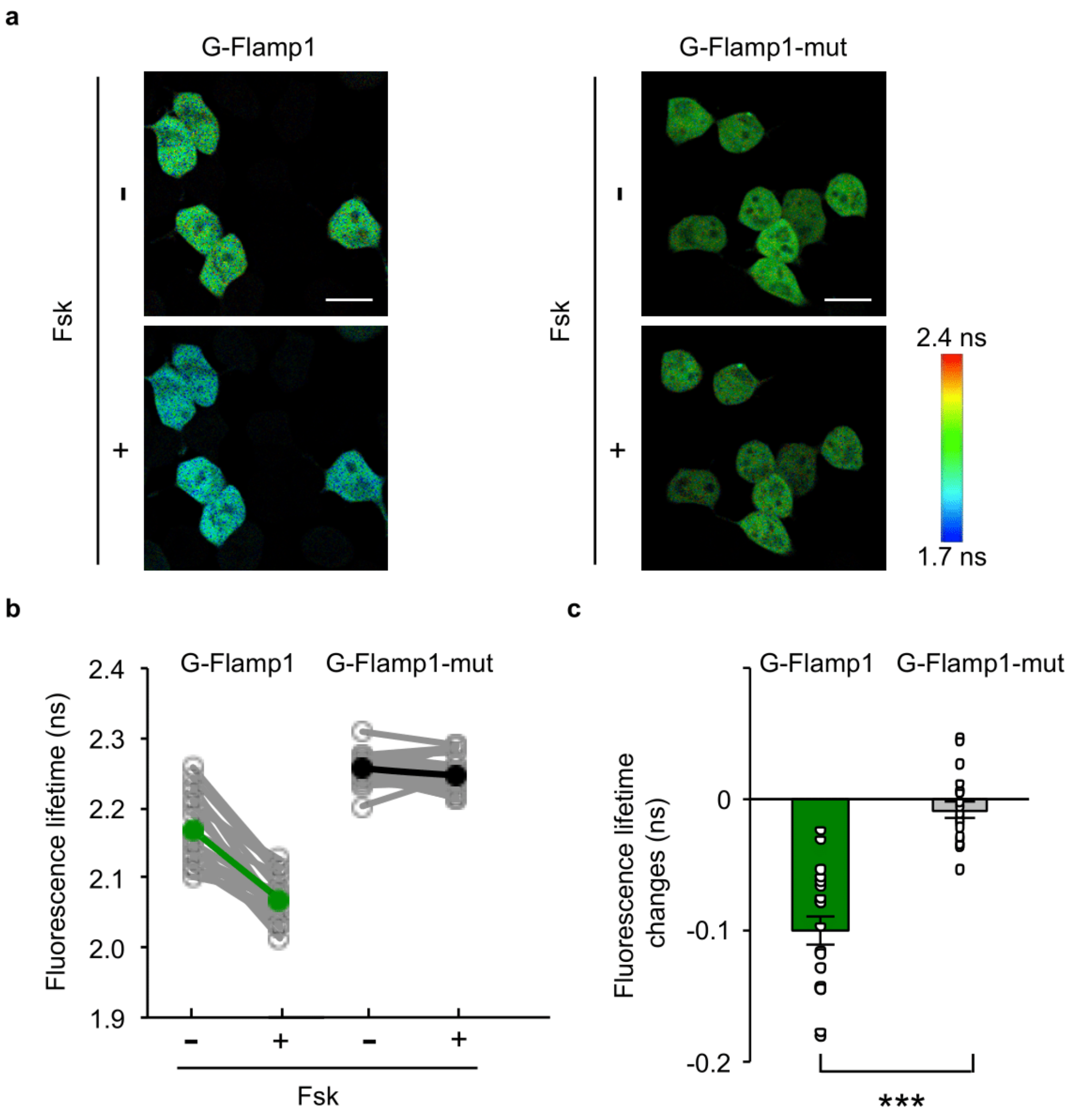

Supplementary Fig. 17 Fluorescence lifetime changes of G-Flamp1 in HEK293T cells.

(a) Representative fluorescence lifetime images of HEK293T cells expressing G-Flamp1 or G-Flamp1-mut before and 10 min after 60 μM Fsk stimulation from 3 independent experiments. The minus and plus signs denote without and with Fsk treatment, respectively. Scale bars: 20 μm.

(b) Summary of the fluorescence lifetimes of G-Flamp1 and G-Flamp1-mut in HEK293T cells before and after 60 μM Fsk treatment. n = 20 cells for both G-Flamp1 and G-Flamp1-mut groups from 3 cultures. Individual data points from single cells and their averages are indicated by gray and green/black circles, respectively.

(c) Fluorescence lifetime changes for G-Flamp1 and G-Flamp1-mut in b. Data are presented as mean ± SEM. Two-tailed Student's *t*-test was performed. $P = 1.3 \times 10^{-8}$ between the two groups. $^{***}P < 0.001$.

Source data are provided as a Source Data file.

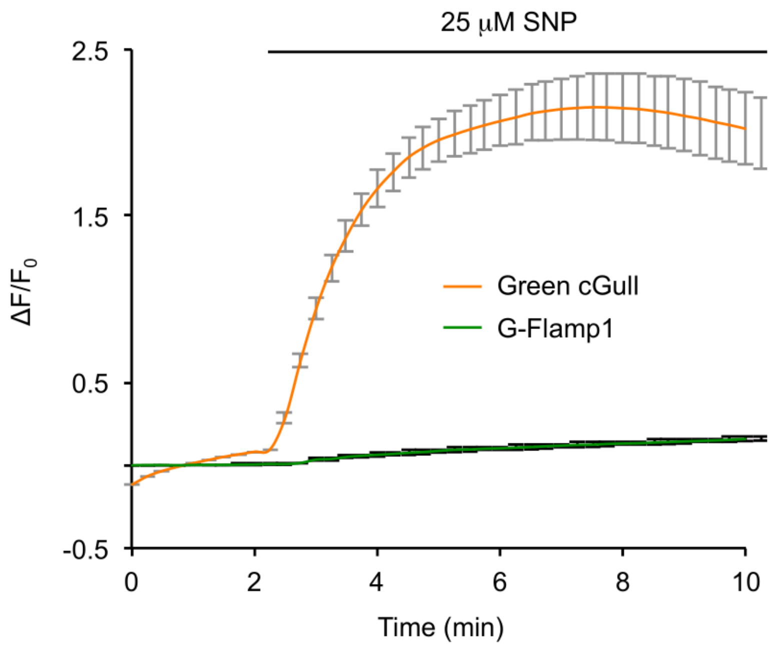

Supplementary Fig. 18 $\Delta F/F_0$ of Green cGull and G-Flamp1 in response to 25 µM SNP in HEK293T cells.

Representative traces of $\Delta F/F_0$ of HEK293T cells expressing Green cGull or G-Flamp1. Data are shown as mean ± SEM. n = 22 cells for Green cGull and n = 15 cells for G-Flamp1 from 3 cultures for both. Source data are provided as a Source Data file.

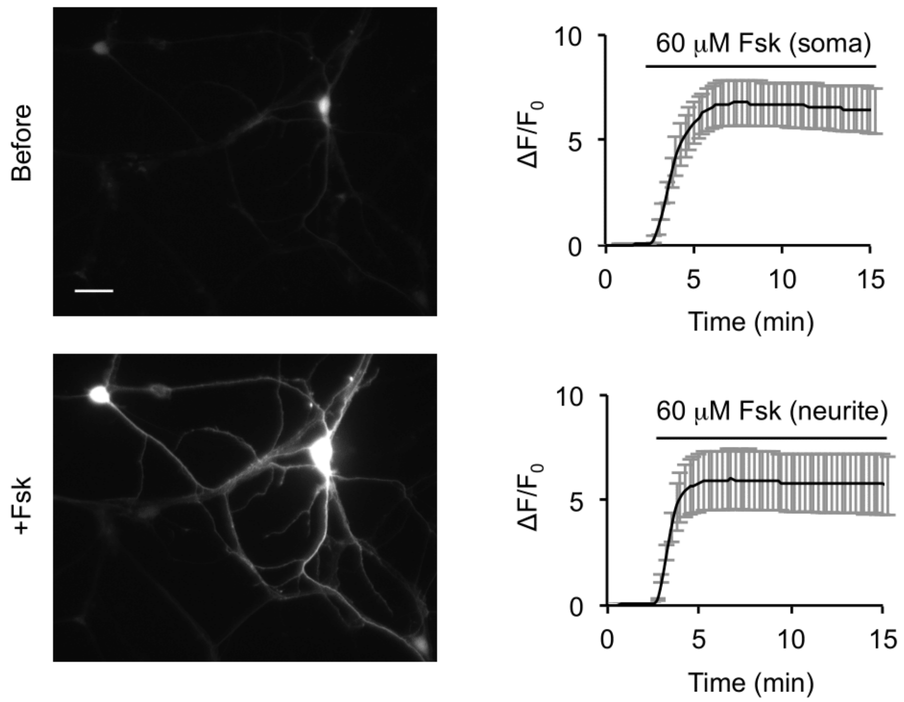

Supplementary Fig. 19 $\Delta F/F_0$ of G-Flamp1 in cultured cortical neurons in response to 60 μM Fsk.

Representative fluorescence images (left) and traces of $\Delta F/F_0$ (right) of cortical neurons expressing G-Flamp1 in response to 60 μM Fsk. Data are shown as mean ± SEM. n = 6 ROIs of 6 neurons for both soma and neurites. Curves are shown as mean ± SEM. Scale bar: 20 μm.

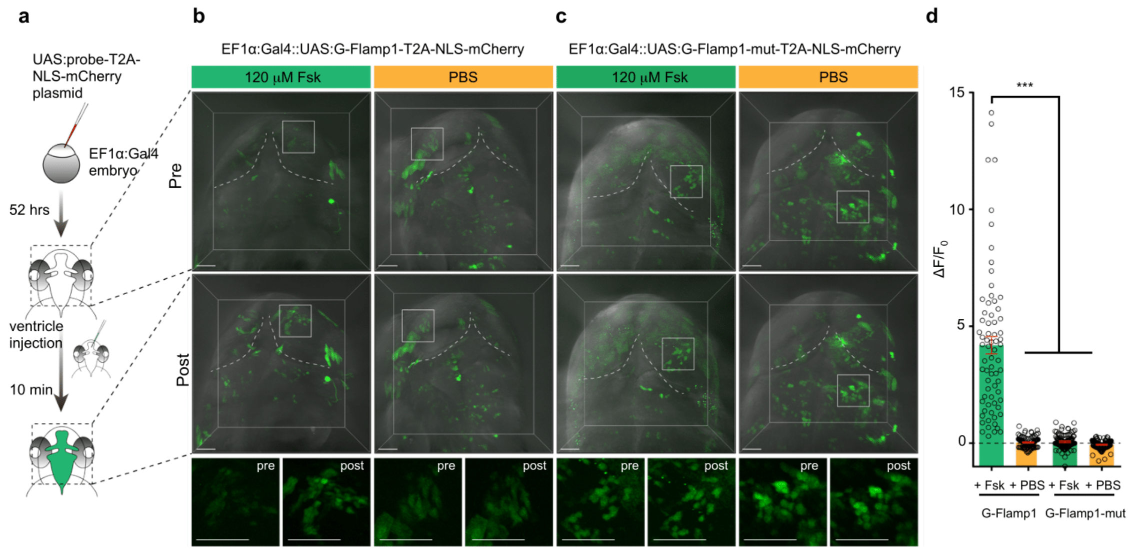

Supplementary Fig. 20 Performance of G-Flamp1 in zebrafish.

(a) Schematic drawing for the experiments in zebrafish.

(b) Representative fluorescent images of G-Flamp1 before and after 120 μM Fsk or PBS injection from 4 (for Fsk) and 3 (for PBS) independent experiments. High-magnification images of the boxed areas are shown below. Scale bars: 50 μm.

(c) Similar as **b** except that G-Flamp1-mut-T2A-NLS-mCherry plasmid was used. Representative fluorescent images of G-Flamp1-mut before and after 120 μM Fsk or PBS injection from 3 (for Fsk) and 3 (for PBS) independent experiments.

(d) Quantification of $\Delta F/F_0$ in the above conditions. Data are shown as mean ± SEM overlaid with data points from individual cells. n = 73 cells from 4 animals for G-Flamp1 with Fsk group, 86 cells from 3 animals for G-Flamp1 with PBS group, 93 cells from 3 animals for G-Flamp1-mut with Fsk group, 92 cells from 3 animals for G-Flamp1-mut with PBS group. Two-tailed Student's *t*-tests were performed. $P = 1.7 \times 10^{-13}$, 2.1×10^{-13} and 7.4×10^{-14} between G-Flamp1 with Fsk group and G-Flamp1 with PBS, G-Flamp1-

mut with Fsk and G-Flamp1-mut with PBS groups, respectively. ***$P < 0.001$. Source data are provided as a Source Data file.

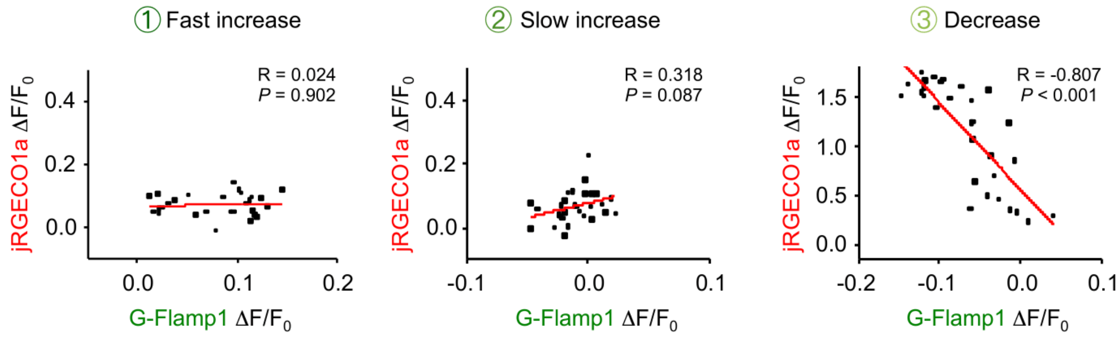

Supplementary Fig. 21 Pearson correlation analysis of time series between G-Flamp1 and jRGECO1a signals in Fig. 4f. Each group includes 30 time points during 0~20 s after running onset. The Pearson's correlation values (R) for the 'fast increase', 'slow increase' and 'decrease' groups were 0.024, 0.318 and -0.807, respectively. Two-tailed Student's *t*-tests were performed and *P* values are also shown. Source data are provided as a Source Data file.

2. Supplementary Tables

Supplementary Table 1 Biophysical and biochemical properties of purified G-Flamp1.

cAMP sensor	Ex/Em[a] (nm), free[b]	Ex/Em (nm), bound[c]	$\Delta F/F_0$ (450 nm)[d]	K_d (μM)[e]	n_H[f]	k_{on} ($\mu M^{-1}s^{-1}$)[g]	k_{off} (s^{-1})[h]	pKa[i], free	pKa, bound	EC[j], free ($M^{-1}cm^{-1}$)	EC, bound ($M^{-1}cm^{-1}$)	QY[k], free	QY, bound
G-Flamp1	500/513	490/510	13.4	2.17	1.13	3.48	7.9	8.27	6.95	4374	25280	0.323	0.322

[a]Excitation peak/Emission peak. [b]cAMP-free form of G-Flamp1. [c]cAMP-bound form of G-Flamp1. [d]Maximum fluorescence change under 450 nm excitation. [e]Dissociation constant. [f]Hill coefficient. [g]Association rate constant. [h]Dissociation rate constant. [i]The pH at which the fluorescence intensity is half-maximal. [j]Extinction coefficient. [k]Quantum yield.

Supplementary Table 2 Molecular brightness and fluorescence change of GCaMP sensors[1].

Sensor	$\Delta F/F_0$[a]	EC[b] × QY[c] ratio (bound/free)	EC, free[d] ($M^{-1}cm^{-1}$)	EC, bound[e] ($M^{-1}cm^{-1}$)	QY, free	QY, bound
GCaMP6f	39	27	2761	66293	0.57	0.66
jGCaMP7s	39	10	5554	53068	0.58	0.65
jGCaMP7c	144	38	1541	49566	0.5	0.59
jGCaMP7b	21	10	5668	56462	0.59	0.6

[a]Maximum fluorescence change. [b]Extinction coefficient. [c]Quantum yield. [d]Ca^{2+}-free form. [e]Ca^{2+}-bound form.

Supplementary Table 3 List of current cAMP indicators[2-5].

Sensor (year[a])	K_d[d] for cAMP (μM)	K_d for cGMP (μM)	$\Delta R/R_0$ or $\Delta F/F_0$[e] (purified sensor)	$\Delta R/R_0$ or $\Delta F/F_0$ (in cells or cell lysate)
CFP-(δDEP,CD)-YFP (2004)[b]	14	Insensitive to cGMP	n.d.	-0.45
^{T}Epac1^{VV}/Epac-S^{H74} (2011)[b]	~10	n.d.	n.d.	~0.82
Epac-S^{H187} (2015)[b]	~4	n.d.	n.d.	~1.6
cAMPFIRE-L (2021)[b]	2.65	n.d.	n.d.	~2.7
cAMPFIRE-M (2021)[b]	1.41	n.d.	n.d.	~3.2
cAMPFIRE-H (2021)[b]	0.38	n.d.	n.d.	~3.3
ICUE1 (2004)[b]	n.d.	n.d.	n.d.	~0.3
ICUE2 (2008)[b]	12.5	n.d.	n.d.	~0.6
ICUE3 (2009)[b]	n.d.	n.d.	n.d.	~1.0
Epac1-camps (2004)[b]	2.35	n.d.	n.d.	~0.24
Epac2-camps300 (2009)[b]	0.3	14	n.d.	~0.8
mlCNBD-FRET (2016)[b]	0.07	0.5	~0.4	~0.47
CUTie (2017)[b]	7.4	n.d.	n.d.	~0.23
cAMPr (2018)[c]	1	No response to 1 mM cGMP	n.d.	~0.5; 0.45[f]
Flamindo2 (2014)[c]	3.2	22	-0.75	-0.7; -0.25[f], -0.75[f,g]
cADDis (2016)[c]	10-100	n.d.	-0.55	n.d.
Pink Flamindo (2017)[c]	7.2	94	3.2	1.30; 0.88[f]
R-FlincA (2018)[c]	0.3	6.6	7.6	6.0; 1.5[f]

[a]Publication year. [b]FRET-based cAMP indicators. [c]Single-FP cAMP indicators. [d]Dissociation constant. [e]The maximum ratio change ($\Delta R/R_0$) and maximum fluorescence change ($\Delta F/F_0$) for FRET sensors and single-FP sensors, respectively. [f]Measured in this study. HEK293T cells were cultured at 37 °C. [g]Value was obtained under two-photon excitation. n.d.: not determined.

Supplementary Table 4 Data collection and structure refinement statistics.

	G-Flamp1 (PDB 6M63)
**Data collection**	
Space group	$P\ 2_1 2_1 2_1$
Cell dimensions	
a, b, c (Å)	87.84, 94.69, 109.99
α, β, γ (°)	90.00, 90.00, 90.00
Resolution (Å)	50.00-2.25 (2.29-2.25)[*]
R_{merge}[**]	0.15 (1.01)
$I / \sigma I$	36.36 (4.45)
Completeness (%)	100.00 (100.00)
Redundancy	14.50 (13.80)
**Refinement**	
Resolution (Å)	47.34-2.25 (2.33-2.25)
No. reflections	43,987 (4,114)
R_{work}[#] / R_{free}[##]	0.18/0.22
No. atoms	5,857
Protein	5,448
Ligand/ion	44
Water	365
B-factors	37.61
Protein	37.16
Ligand/ion	28.42
Water	45.50
R.m.s. deviations	
Bond lengths (Å)	0.007
Bond angles (°)	0.900
Ramachandran favored (%)	98.15
Ramachandran allowed (%)	1.85
Ramachandran outliers (%)	0.00

[*]Statistics for the highest-resolution shell are shown in parentheses.

[**]Rmerge = $\sum_{hkl} \sum_i |I_i\ (hkl) - \langle I(hkl) \rangle| / \sum_{hkl} \sum_i I_i(hkl)$, where $I_i(hkl)$ is the intensity measured for the i th reflection and $\langle I(hkl) \rangle$ is the average intensity of all reflections with indices hkl.

[#]R-work = $\sum_{hkl} ||F_{\mathrm{obs}}\ (hkl)| - |F_{\mathrm{calc}}\ (hkl)|| / \sum_{hkl} |F_{\mathrm{obs}}(hkl)|$.

[##]R-free is calculated in an identical manner using 10% of randomly selected reflections that were not included in the refinement.

Supplementary Table 5 Key parameters for fluorescence imaging data collection.

Figure	Cell	Indicator or FP	Microscope	Objective	Excitation	Emission	Frame interval
2a	HEK293T	GCaMP6s, cAMPr, Flamindo2, G-Flamp1	IX83	20 × 0.75 NA	480/30 nm	530/30 nm	-
2c	HEK293T	G-Flmap1, G-Flamp1-mut	IX83	60 × 1.35 NA	441/20 nm	530/30 nm	15 s
2d-e	HEK293T	G-Flamp1	IX83	60 × 1.35 NA	441/20 nm	530/30 nm	15 s
2d-e	HEK293T	cAMPr, Flamindo2	IX83	60 × 1.35 NA	480/30 nm	530/30 nm	15 s
2d-e	HEK293T	Pink Flamindo, R-FllincA	IX83	60 × 1.35 NA	568/20 nm	630/50 nm	15 s
2f-g	HEK293T	G-Flamp1	IX83	60 × 1.35 NA	441/20 nm	530/30 nm	15 s
2h-i	Cultured mouse cortical neurons	G-Flamp1	IX83	20 × 0.75 NA	441/20 nm	530/30 nm	15 s
3d-f	Fly Kenyon cells	G-Flamp1, GFP	Olympus FV1000	25 × 1.05 NA	930 nm	495-540 nm	0.15 s (odor puff)<br>0.15 s (electrical shock)<br>1 s (Fsk perfusion)
4b-d	Mouse cortical neurons *in vivo*	G-Flamp1, G-Flamp1-mut	Bruker Ultima Investigator	16 × 0.8 NA	920 nm	490-560 nm	0.67s
4b-d	Mouse cortical neurons *in vivo*	jRGECO1a	Bruker Ultima Investigator	16 × 0.8 NA	920 nm	570-620 nm	0.67s
S1b	HEK293T	cAMPr, Flamindo2	IX83	60 × 1.35 NA	480/30 nm	530/30 nm	15 s
S1b	HEK293T	Pink Flamindo, R-FlincA	IX83	60 × 1.35 NA	568/20 nm	630/50 nm	15 s
S1d	HEK293T	R-FlincA	IX83	60 × 1.35 NA	568/20 nm	630/50 nm	-
S12a	HEK293T	Flamindo2, cAMPr, G-Flamp1	Nikon-TI two-photon microscope	25 × 1.4 NA	920 nm	495-532 nm	-
S12b-d	HEK293T	Flamindo2, cAMPr, G-Flamp1	Nikon-TI two-photon microscope	25 × 1.4 NA	920 nm	495-532 nm	5 s
S14a	HeLa	G-Flamp1	IX83	60 × 1.35 NA	441/20 nm	530/30 nm	15 s
S14b	CHO	G-Flamp1	IX83	60 × 1.35 NA	441/20 nm	530/30 nm	15 s
S16	HEK293T	G-Flamp1	IX83	60 × 1.35 NA	480/30 nm	530/30 nm	15 s
S16	HEK293T	G-Flamp1	IX83	60 × 1.35 NA	441/20 nm	530/30 nm	15 s
S17	HEK239T	G-Flamp1, G-Flamp1-mut	Leica TSC SP8 two-photon microscope	25 × 0.95 NA	920 nm	495-550 nm	-
S18	HEK293T	G-Flamp1	IX83	60 × 1.35 NA	441/20 nm	530/30 nm	15 s
S18	HEK293T	Green cGull	IX83	60 × 1.35 NA	480/30 nm	530/30 nm	15 s
S19	Cultured mouse cortical neurons	G-Flamp1	IX83	20 × 0.75 NA	441/20 nm	530/30 nm	15 s
S20b-d	Zebrafish cells *in vivo*	G-Flamp1, G-Flamp1-mut	Olympus BX61WI two-photon microscope	25 × 1.05 NA	960 nm	495-540 nm	1 s

3. Supplementary References

1. Dana, H. et al. High-performance calcium sensors for imaging activity in neuronal populations and microcompartments. *Nature Methods* **16**, 649-657 (2019).
2. Jiang, J.Y., Falcone, J.L., Curci, S. & Hofer, A.M. Interrogating cyclic AMP signaling using optical approaches. *Cell Calcium* **64**, 47-56 (2017).
3. Klausen, C., Kaiser, F., Stüven, B., Hansen, J.N. & Wachten, D. Elucidating cyclic AMP signaling in subcellular domains with optogenetic tools and fluorescent biosensors. *Biochemical Society Transactions* **47**, 1733-1747 (2019).
4. Dikolayev, V., Tuganbekov, T. & Nikolaev, V.O. Visualizing Cyclic Adenosine Monophosphate in Cardiac Microdomains Involved in Ion Homeostasis. *Front Physiol* **10**, 1406 (2019).
5. Massengill, C.I. et al. Highly sensitive genetically-encoded sensors for population and subcellular imaging of cAMP in vivo. *Preprint at bioRxiv* https://www.biorxiv.org/content/10.1101/2021.08.27.457999v1 (2021).
