# Supplementary material for: A high-performance genetically encoded fluorescent indicator for in vivo cAMP imaging
Source: Nat Commun. 2022 Sep 12;13:5363. doi: 10.1038/s41467-022-32994-7 (PMC9468011; doi:10.1038/s41467-022-32994-7)
Supplement: Supplementary file 2 — Reporting Summary [file 41467_2022_32994_MOESM2_ESM.pdf]

nature research

Corresponding author(s): Jun Chu

Last updated by author(s): Aug 4, 2022

Reporting Summary

Nature Research wishes to improve the reproducibility of the work that we publish. This form provides structure for consistency and transparency in reporting. For further information on Nature Research policies, see our Editorial Policies and the Editorial Policy Checklist.

Statistics

For all statistical analyses, confirm that the following items are present in the figure legend, table legend, main text, or Methods section.

n/a	Confirmed	
☐	☒	The exact sample size (*n*) for each experimental group/condition, given as a discrete number and unit of measurement
☐	☒	A statement on whether measurements were taken from distinct samples or whether the same sample was measured repeatedly
☐	☒	The statistical test(s) used AND whether they are one- or two-sided<br>*Only common tests should be described solely by name; describe more complex techniques in the Methods section.*
☒	☐	A description of all covariates tested
☐	☒	A description of any assumptions or corrections, such as tests of normality and adjustment for multiple comparisons
☐	☒	A full description of the statistical parameters including central tendency (e.g. means) or other basic estimates (e.g. regression coefficient) AND variation (e.g. standard deviation) or associated estimates of uncertainty (e.g. confidence intervals)
☐	☒	For null hypothesis testing, the test statistic (e.g. F, t, r) with confidence intervals, effect sizes, degrees of freedom and P value noted<br>*Give P values as exact values whenever suitable.*
☒	☐	For Bayesian analysis, information on the choice of priors and Markov chain Monte Carlo settings
☒	☐	For hierarchical and complex designs, identification of the appropriate level for tests and full reporting of outcomes
☐	☒	Estimates of effect sizes (e.g. Cohen's *d*, Pearson's *r*), indicating how they were calculated

Our web collection on statistics for biologists contains articles on many of the points above.

Software and code

Policy information about availability of computer code

Data collection	1. Micro-manager 1.4.21 of home-made fluorescence system for sensor screening on LB plates.<br>2. Tecan i-control 1.11 of Infinite M1000 fluorometer (Tecan) for absorbance and fluorescence spectra.<br>3. Pro-data Chirascan 4.5.1840.0 of Chirascan spectrometer equipped with an SX20 Stopped-Flow accessory (Applied Photophysics Ltd) for binding kinetics measurement.<br>4. Beamline BL17B1 of the Shanghai Synchrotron Radiation Facility (SSRF) for X-ray diffraction data collection.<br>5. Maestro Version 13.0.135, MMshare version 5.6.135 for Metadynamics molecular dynamics simulations.<br>6. Micro-manager 1.4.21 of IX83 microscope (Olympus) for one-photon imaging of cultured cells.<br>7. NIS-Elements AR 4.30.01 of Nikon-TI two-photon microscope (Nikon) for two-photon imaging of cultured cells.<br>8. Image Lab 6.0.1 Build 34 software of ChemiDoc MP imaging system (Bio-Rad) for western blotting.<br>9. FV10-ASW 4.2 of BX61WI two-photon microscope (Olympus) for two-photon imaging in zebrafish.<br>10. Fluoview 3.1a of FV1000MPE two-photon microscope (Olympus) for two-photon imaging of transgenic flies.<br>11. Prairie View 5.5.64.100 of Bruker Investigator two-photon microscope (Bruker) for two-photon imaging in mice.<br>12. Custom MATLAB and Arduino programs for fiber photometry recording (codes are available upon request and have been used in the following paper: Sun, F. et al. A Genetically Encoded Fluorescent Sensor Enables Rapid and Specific Detection of Dopamine in Flies, Fish, and Mice. Cell 174, 481-496.e419 (2018).)<br>13. Leica Application Suite X 3.5.6.21594 of Leica TSC SP8 two-photon microscope for fluorescence lifetime imaging of cultured cells.
Data analysis	1. ImageJ 1.52p (NIH).<br>2. Matlab R2020a (MathWorks).<br>3. OriginPro 9.1 (OriginLab).<br>4. A motion correction algorism (EZcalcium).<br>5. A linear unmixing algorithm (https://imagej.nih.gov/ij/plugins/docs/SpectralUnmixing.pdf).

6. HKL3000 v716.1, Phaser 2.7.16, Phenix program suite 1.17.1 and Coot 0.9 for crystal structure determination.
7. Maestro Version 13.0.135, MMshare version 5.6.135 for analysis of MD simulation data, PyMOL 2.4.2 for structural analysis.
8. LAS X FLIM/FCS version 3.5.6 for fluorescence lifetime analysis.

For manuscripts utilizing custom algorithms or software that are central to the research but not yet described in published literature, software must be made available to editors and reviewers. We strongly encourage code deposition in a community repository (e.g. GitHub). See the Nature Research guidelines for submitting code & software for further information.

Data

Policy information about availability of data

All manuscripts must include a data availability statement. This statement should provide the following information, where applicable:
- Accession codes, unique identifiers, or web links for publicly available datasets
- A list of figures that have associated raw data
- A description of any restrictions on data availability

The atomic coordinates and structure factors of the G-Flamp1 (no RSET peptide) and cAMP complex have been deposited in the Protein Data Bank (http://www.rcsb.org) with PDB ID code 6M63. Other PDB files used in this study are: 1VP6, 3WLD, 3EVP, 3CLP, 1RL3, 1U12, 2BYV, 4JV4, 3CF6, 6DGV and 5UKG. Plasmids expressing G-Flamp1 and G-Flamp1-mut have been deposited to Addgene (http://addgene.org, cat. nos. 188567, 188568, 188569 and 188570). Source data are provided with this paper.

Field-specific reporting

Please select the one below that is the best fit for your research. If you are not sure, read the appropriate sections before making your selection.

☒ Life sciences ☐ Behavioural & social sciences ☐ Ecological, evolutionary & environmental sciences

For a reference copy of the document with all sections, see nature.com/documents/nr-reporting-summary-flat.pdf

Life sciences study design

All studies must disclose on these points even when the disclosure is negative.

Sample size	No statistical sample-size calculation was performed. Experiments were performed in triplicate unless otherwise noted, which is similar to the literatures in the sensor field.<br>1. Tian, L. et al. Imaging neural activity in worms, flies and mice with improved GCaMP calcium indicators. Nature Methods 6, 875-881 (2009).<br>2. Sun, F. et al. A Genetically Encoded Fluorescent Sensor Enables Rapid and Specific Detection of Dopamine in Flies, Fish, and Mice. Cell 174, 481-496.e419 (2018).<br>3. Patriarchi, T. et al. Ultrafast neuronal imaging of dopamine dynamics with designed genetically encoded sensors. Science 360 (2018).<br>4. Sun, F. et al. Next-generation GRAB sensors for monitoring dopaminergic activity in vivo. Nature Methods 17, 1156-1166 (2020).<br>5. Patriarchi, T. et al. An expanded palette of dopamine sensors for multiplex imaging in vivo. Nature Methods 17, 1147-1155 (2020).
Data exclusions	No data were excluded from the analysis.
Replication	Experiments were performed in triplicate unless otherwise noted. The exact replication number of each experiment is indicated in the respective figure legend. All attempts at replication were successful.
Randomization	Cells and animals were randomly allocated into experiments groups.
Blinding	Blinding is not necessary and was not used in this study. The experimental condition were obvious to the researchers and the analyses were performed objectively and not subjective to human bias.

Reporting for specific materials, systems and methods

We require information from authors about some types of materials, experimental systems and methods used in many studies. Here, indicate whether each material, system or method listed is relevant to your study. If you are not sure if a list item applies to your research, read the appropriate section before selecting a response.

Materials & experimental systems

n/a	Involved in the study	
☐	☒	Antibodies
☐	☒	Eukaryotic cell lines
☒	☐	Palaeontology and archaeology
☐	☒	Animals and other organisms
☒	☐	Human research participants
☒	☐	Clinical data
☒	☐	Dual use research of concern

Methods

n/a	Involved in the study	
☒	☐	ChIP-seq
☒	☐	Flow cytometry
☒	☐	MRI-based neuroimaging

Antibodies

Antibodies used	Primary antibody:<br>(1) Rabbit CREB antibody 48H2 (1:1000) (Signaling Technology, Inc., Cat. No. 9197S)<br>(2) Rabbit Phospho-CREB (Ser133) antibody 87G3(1:1000) (Signaling Technology, Inc., Cat. No. 9198S)<br><br>Secondary antibody:<br>Goat Anti-Rabbit IgG H&L (HRP) (1:3000)(Abcam, Cat. No. ab6721)
Validation	All antibodies were validated either by the manufacturer or used extensively in previously published papers.<br>(1) Rabbit CREB antibody 48H2: the manufacturer website states that this antibody has been validated in SK-N-MC, COS, NIH/3T3, C6 and Drosophila S2 cells.<br>(2)Rabbit Phospho-CREB (Ser133) antibody 87G3:the manufacturer website states that this antibody has been validated in SK-N-MC cells.<br>Both 48H2 and 87G3 antibodies were used in immunoblot of human intestinal epithelial cell (IEC) line SKCO15 in the following paper: www.pnas.org/cgi/doi/10.1073/pnas.1921335117

Eukaryotic cell lines

Policy information about cell lines

Cell line source(s)	HEK293T (ATCC, cat. no. CRL-3216), HeLa (ATCC, cat. no. CCL-2), CHO (ATCC, cat. no. CRL-61).
Authentication	The cells were authenticated based on the morphology under microscope and growth rate.
Mycoplasma contamination	The cell lines were not tested for mycoplasma contamination.
Commonly misidentified lines<br>(See ICLAC register)	No misidentified cell lines were used in this study.

Animals and other organisms

Policy information about studies involving animals; ARRIVE guidelines recommended for reporting animal research

Laboratory animals	1. E16 BALB/c mice (male and female) (Beijing Vital River Laboratory Animal Technology) for cortical neuron preparation.<br>2. Larval zebrafish 52 hours post-fertilization (male and female) were used for two-photon imaging.<br>3. Adult female transgenic Drosophila lines within 2 weeks after eclosion were used for in vivo two-photon imaging.<br>4. Adult (P42-90) female wild-type C57BL/6J mice (Beijing Vital River Laboratory Animal Technology) for in vivo two-photon imaging.<br>5. Adult (P60-150) male wild-type C57BL/6N (Shanghai LingChang Experiment Animal Co., Ltd) for fiber photometry recording.
Wild animals	No wild animals were used in this study.
Field-collected samples	No filed-collected samples were used in this study.
Ethics oversight	The animal experiments were performed using protocols that were approved by the Institutional Animal Care and Use Committees at Shenzhen Institute of Advanced Technology-CAS, Peking University, and Institute of Neuroscience-CAS.

Note that full information on the approval of the study protocol must also be provided in the manuscript.
